# Nano-structured strategies in combatting neurodegeneration

**DOI:** 10.3389/fbioe.2025.1638668

**Published:** 2025-12-02

**Authors:** Mani Iyer Prasanth, Anjali R. Mallya, William C. Cho, Deepa Mundekkad

**Affiliations:** 1 Division of Molecular Genetics and Cancer, NITTE University Centre for Science Education and Research (NUCSER), Nitte (Deemed to be University), Mangaluru, Karnataka, India; 2 Department of Biotechnology, Nehru Arts and Science College, Coimbatore, Tamil Nadu, India; 3 Department of Clinical Oncology, Queen Elizabeth Hospital, Hong Kong SAR, China

**Keywords:** Parkinson’s disease, dopaminergic neurons, nano-structured technologies, nanomedicine, targeted drug delivery, neuroprotection, neurodegeneration, progressive neurodegenerative disorder

## Abstract

Parkinson’s disease (PD) is a progressive neurodegenerative disorder that is characterized by the loss of dopaminergic neurons, leading to severe motor and cognitive impairments. Recent advancements in nanomedicine and nano-structured technologies have opened new avenues for targeted drug delivery and neuroprotection, improving therapeutic efficacy and diagnostic accuracy. By harnessing innovative nanotechnological platforms, researchers aim to enhance clinical trial outcomes and refine early-stage diagnostic advancements, offering hope for improved disease management. However, since the pathophysiology of PD is diverse, there are limited treatment options available. This review explores the potential of the recent nanostructured technologies in managing the complexities of PD. Deliberations on the insights from nanomedicine, neurobiology, and material science, on how these emerging and technologically sound nanostructured approaches help in the prevention, diagnosis and treatment of PD will be discussed. Further, the role of nanocarriers in targeted drug delivery involving nanoscale materials specifically for neuroprotection and regeneration will be discussed with special emphasis on the role of nanotechnology in advancing diagnostic methodologies. Additionally, we aim to chart a course for future research directions, with special reference to innovative approaches in disease diagnosis. The various therapeutic approaches, along with the ongoing clinical trials and real-world applications, are expected to add value to the efforts of the researchers worldwide to enhance therapeutic efficacy and patient outcomes in PD.

## 1 Introduction

**FIGURE 1:**
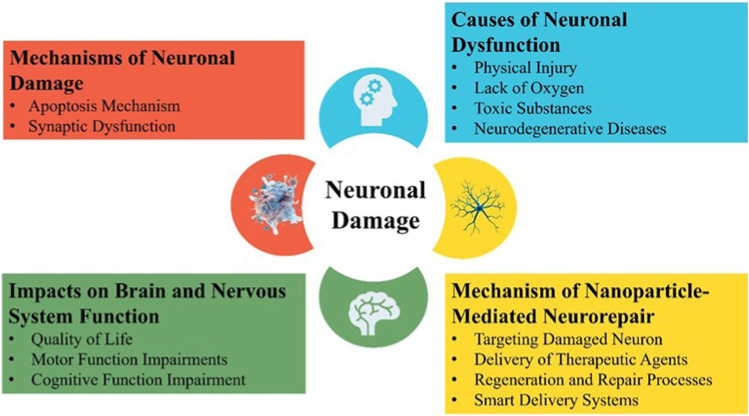
Neuronal damage. The deterioration or loss of nerve cells (neurons) due to factors like oxidative stress, neuroinflammation, toxic accumulation, or trauma, leads to neuronal damage resulting in impaired brain function. It is commonly associated with neurodegenerative diseases such as Parkinson’s, Alzheimer’s, and multiple sclerosis, affecting cognition, movement, and overall neurological health.

Worldwide, millions of individuals, along with their families, suffer due to the health challenges posed by the neurodegenerative diseases (NDs) like Alzheimer’s disease (AD), Parkinson’s disease (PD), and multiple sclerosis. Most of these conditions feature a progressive degeneration of the nervous system, that will ultimately lead to severe decline/dysfunction of cognitive and motor systems thereby impairing the quality of the patient’s life. The situation is made serious with the concurrent immune dysfunction that has a critical role to play in the pathogenesis of many of these NDs, in addition to abnormal immune responses (Zhang et al., 2023). This combined effect can lead to severe neuronal damage that can in turn, exacerbate the disease progression (Figure 1).

PD ranks second as the most common neurodegenerative disease all over the world, with a rise in global prevalence up to 74.3% between 1990 and 2016. The first authentic publication about the disease was done by James Parkinson, from whom the disease got the name, in 1817, followed by many more (Dong-Chen et al., 2023). PD is a chronic and progressive neurological disorder wherein the neurons in the substantia nigra become damaged, which will lead to a reduction in the dopamine levels in the brain, resulting in a gradual loss of regulation in movement and co-ordination. The major symptoms observed are related to the motor movements including tremor, muscle stiffness, loss of balance along with anxiety, depression and sleep-related disorders. The traditional taxonomy of PD includes several subtypes, such as tremor-dominant and non-tremor-dominant PD. Patients with the tremor-dominant subtype generally experience slower disease progression, better cognitive function, fewer non-motor symptoms, lower rates of death and disability, and longer survival compared to those with non-tremor-dominant PD (Zetusky et al., 1985; Marras and Chaudhuri, 2016).

The research involving nanotechnology to better understand, diagnose, and treat PD is emerging as a promising frontier. By utilizing nanoscale materials and carriers, researchers can develop highly targeted drug delivery systems that cross the blood-brain barrier (BBB), ensuring that therapeutics reach the intended regions of the brain with precision. This approach not only enhances the efficacy of neuroprotective agents but also minimizes side effects, offering new credence for more effective management of this complex neurodegenerative disorder. Additionally, nanotechnology facilitates the development of advanced diagnostic tools that can detect PD at earlier stages, potentially enabling timely intervention and improved patient outcomes (Yadav et al., 2025). The mechanisms highlighting the versatility of nanomaterials in addressing various aspects of neuronal damage, are explained in Table 1.

**TABLE 1:** Diverse mechanisms highlighting the versatility of nanomaterials in addressing various aspects of neuronal damage, making them promising candidates for therapeutic interventions in neurodegenerative diseases.

S. No.	Mode of intervention	Mechanism of action	Examples	References
1	Antioxidant activity	scavenges reactive oxygen species (ROS) and reduces oxidative stress	gold and silver nanoparticles have shown significant antioxidant properties	Soliman et al. (2023)
2	Anti-inflammatory activity	modulate neuroinflammation, inhibiting the activation of microglia and astrocytes, reduce the release of pro-inflammatory cytokines	silica nanoparticles have demonstrated the ability to suppress inflammatory responses	Hou et al. (2022)
3	Drug delivery systems	improve the bioavailability and efficacy of neuroprotective drugs, enhance their delivery across the BBB	liposomes, polymeric nanoparticles, and micelles are used for targeted delivery	Correia et al. (2022), Achar et al. (2021)
4	Neurotrophic factor delivery	deliver neurotrophic factors, such as brain-derived neurotrophic factor (BDNF), promotes survival and regeneration of neuronal cells	poly(lactic-co- glycolic acid) (PLGA) nanoparticles have been engineered to encapsulate and sustainably release BDNF, aiding in neuroprotection	Seyedebrahimi et al. (2021), Wu et al. (2022), Bondarenko and Saarma (2021)
5	Neuroprotection via gene delivery	serve as carriers for gene therapy, delivers therapeutic genes that promote neuronal survival, inhibit pathways leading to neurodegeneration	PAMAM dendrimers provide efficient delivery of genetic material, reducing toxicity and improving neuronal targeting	Pérez-Carrión and Posadas (2023), Li et al. (2022)
6	Regeneration of neural tissue	mimic the extracellular matrix, provide a supportive environment for neuronal growth and regeneration, facilitate repair after injury	nanofibers and scaffolds	Du et al. (2023)
7	Enhanced cellular uptake	enhance uptake of therapeutic agents by neuronal cells, improve the overall efficacy of these therapeutic agents	superparamagnetic iron oxide nanoparticles (SPIONs) can be guided via external magnetic fields with improved cellular uptake and enhanced targeting efficiency	Wei et al. (2021), Guigou et al. (2021)
8	Mimicking biological systems	enhancing cellular communication and function, beneficial in restoring neuronal health	biomimetic nanoparticles designed to imitate extracellular vesicles help facilitate neuron-to-neuron communication	Meng et al. (2022), Zhong et al. (2023)
9	Ion channel modulation	may interact with ion channels in neuronal membranes, influence neurotransmission and neuronal excitability	graphene-based materials can impact ion channel activities	Wei and Wang (2021), Kumar et al. (2021)
10	Photothermal and photodynamic effects	be activated by light to induce localized heating (photothermal therapy), produce reactive species (photodynamic therapy) to promote cellular repair, selectively induce apoptosis in diseased cells	upconversion nanoparticles convert low-energy NIR light into high-energy UV/visible light, activating therapeutic agents for both photothermal and photodynamic effects	Dash et al. (2024), Zeng et al. (2021)
11	Chemo- and biodegradability	therapeutic agents are slowly released over time, provide prolonged protection and support to neuronal cells, minimize toxicity	hydrogel nanoparticles provide slow and controlled drug release while maintaining biocompatibility	Omidian and Dey Chowdhury (2025), Pal et al. (2024)
12	Inhibition of protein aggregation	stabilize proteins and prevent the aggregation associated with neurodegenerative diseases, such as amyloid-beta in Alzheimer’s disease	functionalized AuNPs can bind amyloid-beta peptides, preventing their self-assembly and aggregation	Zhao et al. (2022), Abbas (2021)
13	Cell signaling modulation	modulate signaling pathways involved in cell survival and apoptosis, influence neuronal cell fate, affect pathways such as MAPK, PI3K/Akt, and NF-kB	polymeric nanoparticles (PLGA, PEGylated nanoparticles) encapsulate anti-inflammatory agents like curcumin, suppressing activation of pro-inflammatory mediators to reduce neuroinflammation	Keshavarz Shahbaz et al. (2024), Rani et al. (2023)

The transient landscape of nanomedicine in PD emphasizes the urgent need for an extensive review of the subtleties in the field to identify future directions. Despite significant progress in modern medicine, treatment options remain limited due to the PD’s intricate pathophysiology. A focused review on nanostructured technologies will help to deliver valuable insights into how these innovations can address existing therapeutic gaps and improve drug delivery efficiency. This will help future scientists to design new techniques for early diagnosis and innovative treatment options. Such an overview is essential to guide ongoing research efforts and to foster collaborative advancements in the field. This review highlights the key advancements in nanotechnology-based strategies for PD, reiterating the role of nanocarriers in targeted neuroprotection and regeneration, as well as novel diagnostic methodologies. It also mentions the ongoing clinical trials and real-world applications, specifying how these cutting-edge approaches are translating from laboratory research into clinical practice. By charting the recent achievements and future research avenues, this review aims to support the development of a more effective and personalized treatment modality that can ultimately improve the quality of life for PD patients worldwide.

## 2 PD–the pathophysiology and underlying mechanisms

**FIGURE 2:**
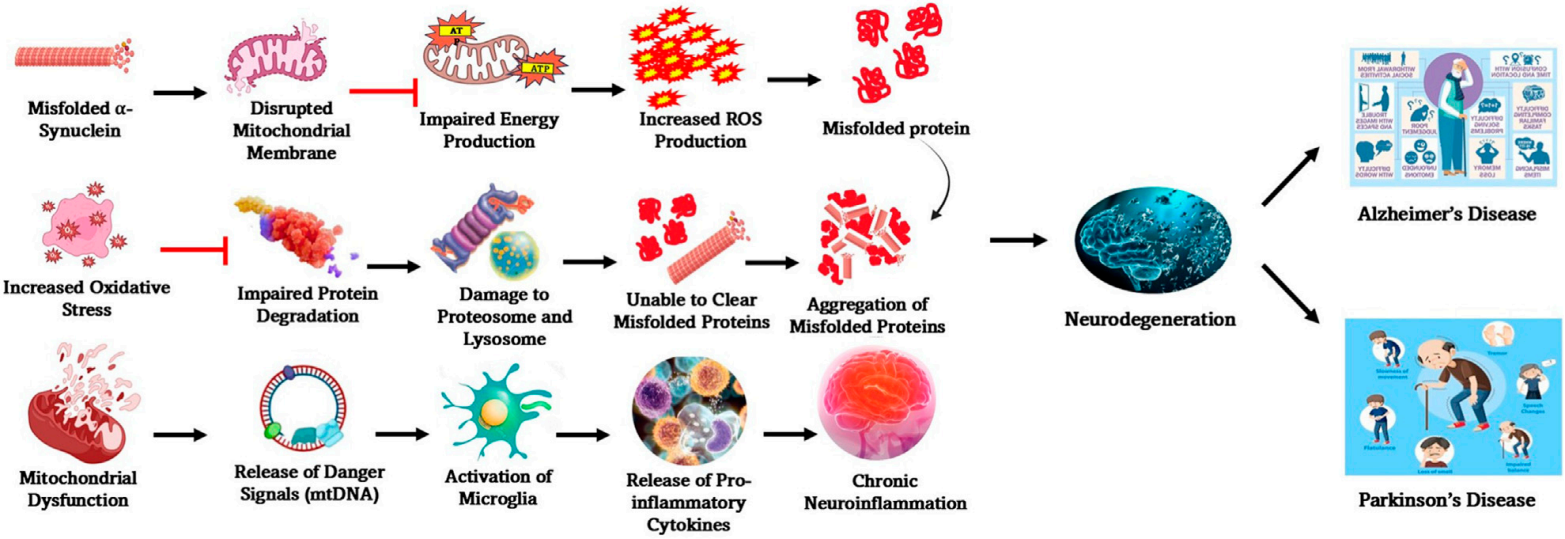
Molecular mechanisms involved in the manifestation of PD. Some of the key processes involved in the interconnected molecular mechanisms underlying PD pathogenesis are α-synuclein aggregation, mitochondrial dysfunction, oxidative stress, impaired protein degradation, and neuroinflammation. Many of these pathways influence one another, amplifying neurodegeneration and contributing to disease progression.

The probability of PD increases significantly post the age of 60, which might go up to over 3% in individuals older than 80 (Pringsheim et al., 2014), wherein men are more susceptible for developing PD than women and dementia can also surface in later stages (Hely et al., 2005). Differences in lifestyle and environmental factors are likely to contribute to the variation in prevalence, observed across regions and ethnic groups. Exposure to toxins from environment may trigger the symptoms of PD, while smoking and caffeine consumption can increase the risk (Dong-Chen et al., 2023). PD progression is characterized by dyskinesia, psychosis, and motor and non-motor fluctuations. Almost 80% of PD patients have freezing of the gait and falls after roughly 17 years of the disease, and up to 50% of patients say they have experienced choking. Many of the initial pathological features of PD point to the gradual degeneration of a specific subset of neurons in the substantia nigra. In the primary phases of the disease, dopaminergic neuron loss is largely confined to the ventrolateral region of the substantia nigra, which further spreads as years pass by. Furthermore, in different areas of the brain, including the cerebellar nuclei and adjacent white matters, certain neurons have abnormally high levels of α-synuclein (Braak et al., 2003). The aggregated α-synuclein accumulates in different neurons, as well as olfactory neurons, forming Lewy bodies (Piao et al., 2003; Mori et al., 2003). The Lewy bodies, a common pathological hallmark, are primarily found in Bergmann glia within the molecular layer and in Purkinje cell axons. As PD progresses, Lewy body accumulation increases, affecting not only dopaminergic neurons but also non-dopaminergic neurons in various brain regions, including the limbic system and neocortex (Hirsch et al., 2003), which further extends to neurons outside the central nervous system (CNS), including the olfactory enteric nervous system (Dong-Chen et al., 2023). More details on the molecular mechanisms involved in the manifestation of PD are explained in Figure 2.

**FIGURE 3:**
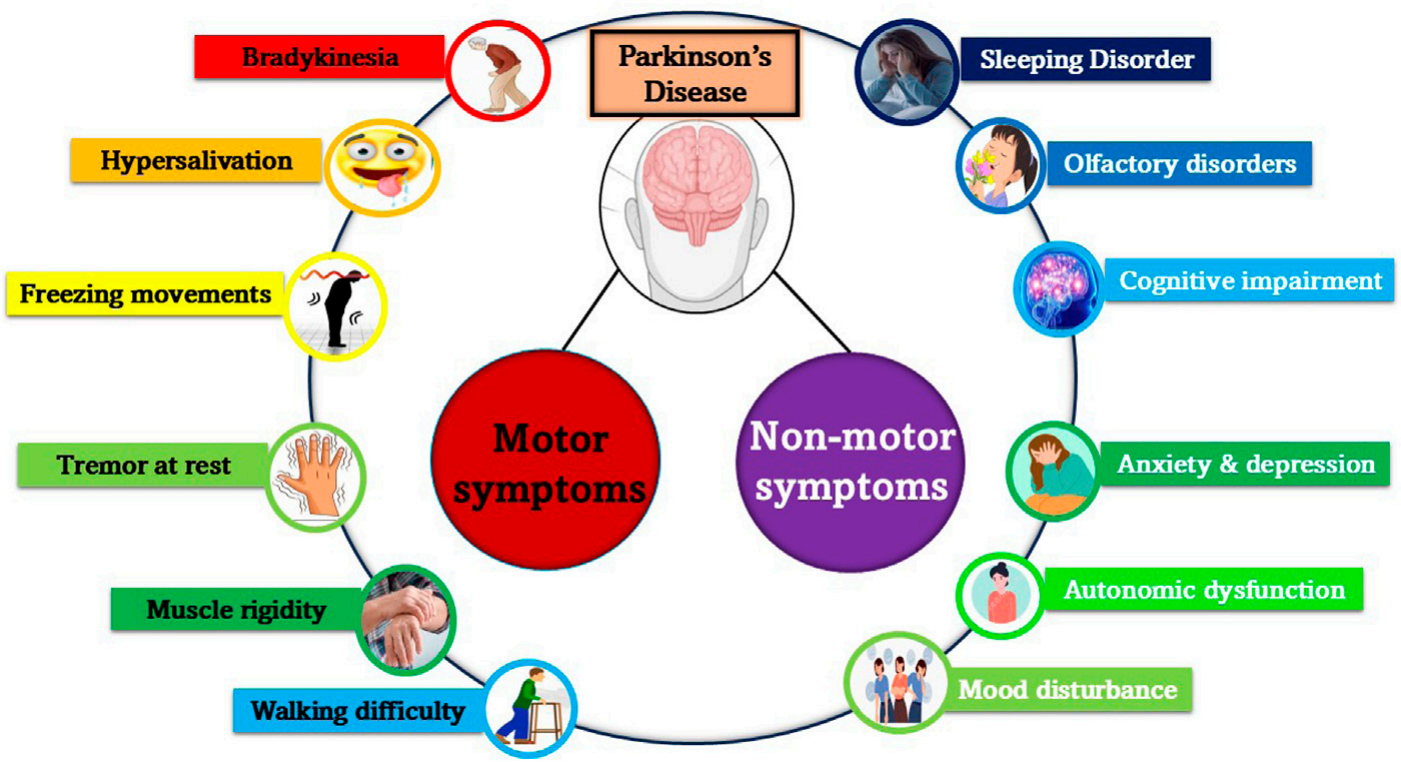
Motor and non-motor symptoms associated with PD. The diverse symptoms of PD are categorized into motor and non-motor manifestations. Motor symptoms primarily affect movement and coordination. Non-motor symptoms significantly impact overall wellbeing and quality of life. Understanding these symptoms is crucial for comprehensive disease management and patient care.

It is difficult to diagnose PD in the early stages. An accurate clinical diagnosis is made in approximately 65% of patients within the first 5 years of PD onset (Sayyaed et al., 2023). However, pathological changes can exist for several years before any noticeable symptoms appear. On average, there is a 12–14-year gap between the initial development of Parkinsonian pathology and the emergence of motor symptoms, illustrating how the preclinical stage of the disease can be prolonged (Greener et al., 2021). However, within the brain, lack of dopamine-producing neurons slows down the motor function considerably, followed by aggregation of lewy bodies (Latif et al., 2021). Environmental contamination including pesticides, or misuse of pills, along with genetic and age factors could initiate the disease, which could potentially aggregate. Cellular aging of neurons in the brain is regulated by inflammatory process over a period of time (Crowley et al., 2019). Additionally, PD patients with resting tremor show reduced grey matter volume primarily in the quadrangular lobe. Compared to those with the akinesia/rigidity-dominant subtype, tremor-dominant PD patients exhibit decreased grey matter volume in the left cerebellar lobule VIIIa, highlighting the relation between cerebellum and PD with tremor (Benninger et al., 2009; Piccinin et al., 2017). However, whether the volumetric change is a causal factor, consequence, or concomitant phenomenon is still a mystery (Zhong et al., 2022). PD progression is marked by worsening motor function that can be managed through symptomatic treatments. In the advanced stages, treatment-resistant motor and non-motor symptoms (Figure 3) become more prominent and axial motor symptoms such as gait disturbances, frequent falls, freezing of gait, speech impairments, and swallowing difficulties are common. Non-motor symptoms in late-stage PD often include symptomatic postural hypotension, persistent constipation requiring regular laxatives, and urinary incontinence (Neag et al., 2020). After 20 years of living with the disease, around 83% of PD patients develop dementia, which significantly contributes to functional decline that need hospitalization and serves as a strong predictor of mortality (Bjørklund et al., 2019).

The common symptoms associated with PD include motor activities like tremors, rigidity, and bradykinesia (slow movements), and non-motor activities like decline of cognitive abilities, mood swings, and dysfunction of the autonomic nervous system, resulting in severe sweating, dizziness and fainting (Bloem et al., 2021; Silva et al., 2023). These multifactorial end-results observed in PD necessitate an intense but holistic approach for managing the obstacles and setbacks associated with this condition. Patients enduring the complex manifestations of this disease depend on the advancements in the field to shed light on the therapeutic strategies that are probably going to be their only way to liberation from the inconveniences associated with this condition. New treatment modalities, including pharmacological and surgical interventions, and supportive therapies need to be discovered to improve the quality of life of the PD patients. It is also important for a precise detection of disease condition as mis-information or incorrect diagnosis will lead to wrong treatment plans that will in turn lead to the worsening of the disease or may result in delayed response in the patient (Wang and Wang, 2023).

### 2.1 Genetic and epigenetic factors affecting the progression of PD

The complexity of PD is intensified by a combination of genetic, epigenetic, and environmental factors. In this, genetic predisposition plays a significant role, with several genes being intimately linked to the risk of PD eventuality. The fact that such genetic mutations can be inherited through autosomal dominant or recessive inheritance patterns further increase the individual’s susceptibility (Ye et al., 2023). Additionally, epigenetic modifications are also known to dominate the progression of PD by regulating genes related to cellular processes like autophagy, inflammation, and oxidative stress (Chen et al., 2022). Understanding the crosstalk between genetic and epigenetic changes in gene expression will also help to uncover potential avenues for early diagnosis, targeted therapies, and personalized interventions. Even though multiple genetic factors contribute to neurodegeneration, mutations in seven major genes such as VPS35, DJ-1, GBA1, LRRK2, PINK1, PRKN, and SNCA impact neuronal health the most through distinct pathways. VPS35 is part of the retromer complex involved in endosomal sorting; a mutation in the VPS35 gene, specifically the D620N mutation, can lead to autosomal-dominant PD. This disrupts the normal functioning of the retromer complex impairing endosomal sorting and protein recycling, further leading to abnormal protein accumulation and neurodegeneration (Rowlands and Moore, 2024). Studies in knock-in mouse models reveal that this mutation also amplifies glutamate synapse activity, contributing to excitotoxicity and neuronal damage (Kadgien et al., 2021). DJ-1 functions as an antioxidative stress protein; its loss heightens oxidative damage to neurons. Mutations to this gene will disrupt its ability to neutralize ROS, making dopaminergic neurons more vulnerable to oxidative damage (Alshammari, 2025). This gene strengthens the mitochondrial function by regulating the uncoupling proteins UCP4 and UCP5, and maintaining mitochondrial membrane potential. Mutations impair this regulation, leading to mitochondrial stress and apoptosis (Mencke et al., 2021). It is also known to interact with Nrf2, a key transcription factor that activates antioxidant response elements (ARE). By failing to regulate redox balance and protein degradation pathways, mutations prevent DJ-1 from promoting Nrf2 nuclear translocation, bringing down the cellular defense against oxidative stress. It can also exacerbate α-synuclein aggregation, a hallmark of PD (Alshammari, 2025). The mutations in GBA1 impair the enzyme glucocerebrosidase (GCase), leading to defective lysosomal degradation and accumulation of toxic α-synuclein aggregates. GCase mutations impair the breakdown of glucosylceramide, leading to lipid accumulation and lysosomal stress. Also, the increased α-synuclein accumulation due to reduced GCase activity leads to the disruption of neuronal function that further accelerates neurodegeneration. Mutations in GBA1 will also contribute to endoplasmic reticulum (ER) stress and mitochondrial dysfunction, further exacerbating neuronal damage (Do et al., 2019; Zhang et al., 2024). Mutations in the LRRK2 gene are among the most common genetic causes of PD (El Otmani et al., 2023). Typically, these mutations lead to increased kinase activity, disrupting cellular processes and contributes to neurodegeneration. Enhanced kinase activity triggers abnormal phosphorylation of Rab proteins, which are crucial for intracellular trafficking. These mutations also impair autophagy and lysosomal degradation, reducing the clearance of α-synuclein. Further, neuronal damage triggers inflammatory pathways, increasing microglial activation. However, unlike the typical PD cases, some LRRK2 mutation carriers exhibit reduced Lewy body accumulation, suggesting alternative neurodegenerative mechanisms (Dzamko, 2025; Rivero-Ríos et al., 2020).

PINK1 and PRKN (Parkin) are two genes heavily invested in mitochondrial quality control through clearance of damaged mitochondria via mitophagy (Mundekkad and Cho, 2022). They are linked to autosomal recessive PD, where two mutated copies of a gene (one from each carrier parent) are needed to cause the disease. Progeny has 50% chances of inheriting the disease and 25% of being a carrier or being devoid of the disease (Antonarakis, 2019). PINK1 detects mitochondrial damage, while Parkin tags and stimulates damaged mitochondria for degradation. Mutations in either of these genes will compromise this process, quickening the accumulation of dysfunctional mitochondria (Ge et al., 2020). The functional loss of these two genes may also lead to consequent events like oxidative stress, energy deficits, and neuronal death, particularly affecting dopaminergic neurons in the substantia nigra (Narendra and Youle, 2024). On account of the high metabolic demands, dopaminergic neurons are particularly susceptible to mitochondrial dysfunction caused by PINK1/Parkin mutations. Genetic mutation in PRKN will contribute to protein aggregation and cellular toxicity which are decisive factors in PD progression. The final of the main seven genes impacting PD, the SNCA gene encodes α-synuclein, and is primarily involved in synaptic vesicle trafficking and release of neurotransmitters. Mutations in this gene (especially as A53T, A30P, and E46K) will catalyze the misfolding and abnormal aggregation of α-synuclein, forming Lewy bodies. Aggregated α-synuclein will obstruct synaptic transmission, mitochondrial function, and protein degradation pathways, contributing to dopaminergic neuron loss. Since α-synuclein aggregates can spread between neurons and to other brain regions, the disease progresses beyond the substantia nigra arousing ultimate damage to the brain cells, disrupting neuronal functions (Meade et al., 2019).

### 2.2 Nanoparticle-based modulation of genetic and epigenetic drivers in PD

**FIGURE 4:**
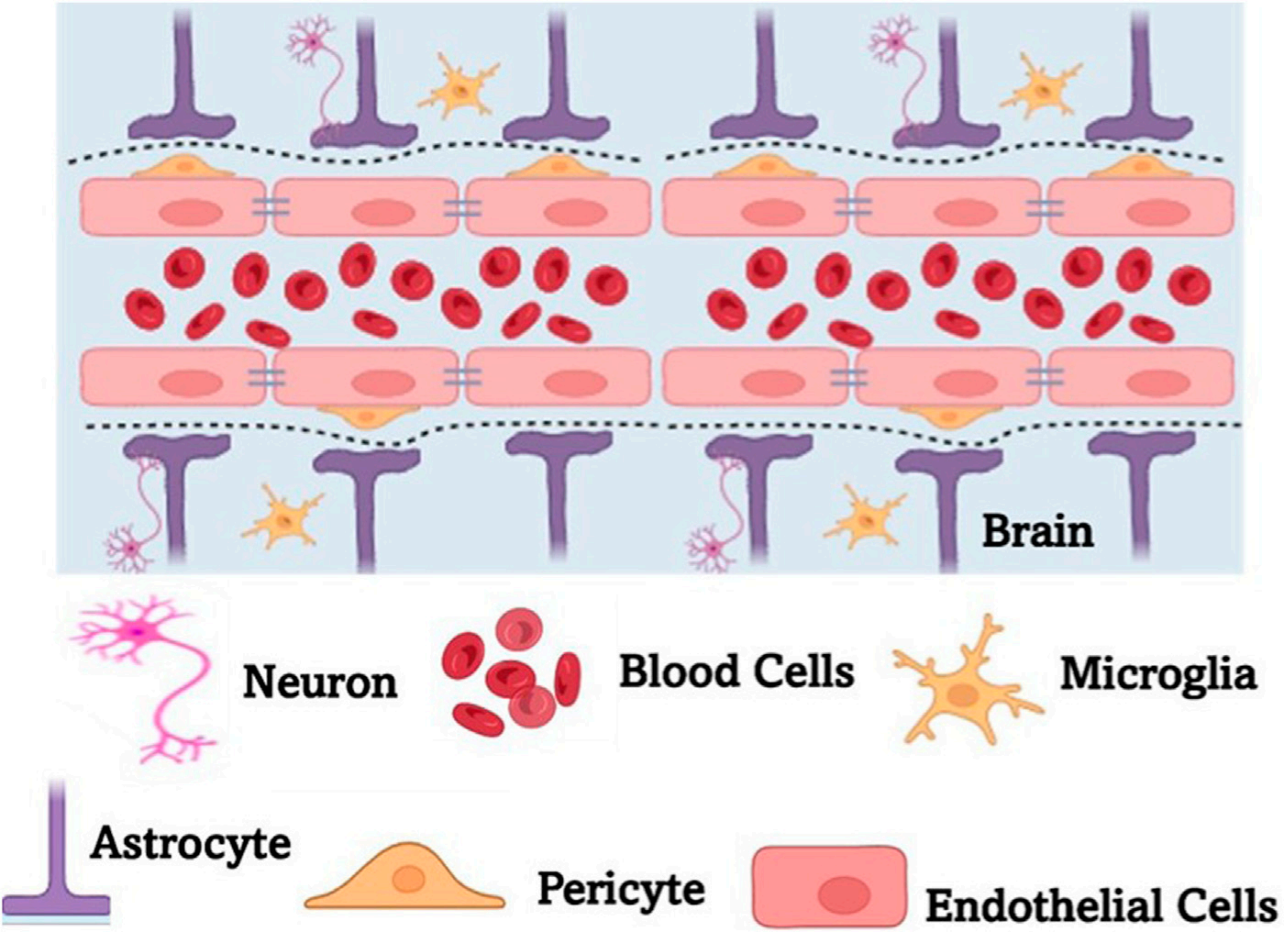
A schematic representation of the blood brain barrier (BBB). BBB is a highly selective, semipermeable membrane formed by tightly packed endothelial cells that regulate the transfer of solutes between the bloodstream and the CNS. BBB protects the brain from harmful substances while allowing essential nutrients to pass through. It plays a crucial role in maintaining brain homeostasis but also poses challenges for drug delivery, requiring specialized strategies to transport therapeutic agents across the barrier.

The precise targeting of genetic as well as epigenetic agents in the progression of PD is well studied in recent years. Nanoparticle-mediated correction of aberrant DNA methylation was found to mitigate neurodegeneration. By the modification of the genes involved in regulating oxidative stress responses, gold nanoparticles, functionalized with S-adenosylmethionine (SAM, a well-studied methyl donor molecule), have demonstrated the ability to restore redox balance and thus modulate epigenetic markers that are closely associated with neurodegeneration in PD (Thanan et al., 2014; Bekdash, 2023). Such interventions at the epigenetic level not only mitigate neurodegeneration but are also known to enhance neuroplasticity and promote repair. Many of the advanced techniques are based on the application of nanoparticles like lipid nanoparticles for the delivery of small interfering RNAs (siRNAs) or antisense oligonucleotides (ASOs) to silence the faulty genes like SNCA that encodes α-synuclein (Cole et al., 2021). It is well known that excessive accumulation of α-synuclein is a hallmark of PD. Cellular delivery of LNP-mediated siRNA has resulted in reduced protein accumulation followed by neurotoxicity in preclinical models. Further, PEGylated LNPs that efficiently encapsulate siRNA can be used against SNCA as they are shown to efficiently penetrate the BBB (Figure 4) and sustain gene knockdown in dopaminergic neurons. Dendrimer-based nanocarriers are also engineered to deliver various components of CRISPR-Cas9 for the targeted editing of LRRK2 mutations that are linked to familial PD (Unnithan et al., 2024). Using the nanocarriers for such targeted delivery ensures high efficiency in transfection with minimal off-target effects. Additionally, the histone acetylation is modulated as part of epigenetic modification – the DNA methylation patterns influence neuroinflammation and, in turn, the survival of neuronal tissues. This is clearly explained by the role of poly (lactic-co-glycolic acid) (PLGA) nanoparticles, which are loaded with suberoylanilide hydroxamic acid (SAHA), a histone deacetylase (HDAC) inhibitor. The acetylation balance is restored by SAHA, which in turn protects the dopaminergic neurons observed in PD models that are induced experimentally by 1-methyl-4-phenyl-1,2,3,6-tetrahydropyridine (MPTP) (Mondal and Firdous, 2025). Such modulations are stimulated through multiple levels of disease-driven molecular pathways with exceptional specificity and biocompatibility. In general, the modulation of genetic and epigenetic modulators by nano-structured interventions signifies a paradigm shift in therapeutics related to NDs.

## 3 Nanomedicine in neurodegenerative diseases

The nanostructured materials are impacting various phases of medical fields, and they are now being applied in neuroprotection, extending to various approaches including the delivery of antioxidant and anti-inflammatory molecules/agents, and for neurotrophic factor supplementation. To elaborate on the use of nanostructured molecules in the medical filed, graphene oxide nanoparticles are proving to be a promising candidate in antioxidant therapy by effectively scavenging free radicals and reducing oxidative stress - these are crucial factors in neurodegenerative diseases like PD. In addition, biomimetic nanofibers, designed to mimic the extracellular matrix (Mundekkad and Mallya, 2025), can support neuronal growth and regeneration, demonstrating potential in neurorepair strategies. Nanomedicine has also introduced many advanced diagnostic tools to be used in NDs along with other advanced biotechnological innovations. In many cases, gold nanoparticles are known to be employed explicitly to bind with specific biomarkers associated with proteins specific to NDs. Specifically, in case of AD, many of these nanoparticles are used in combination with other imaging processes to detect the proteins that are specifically found in NDs like the beta-amyloid or tau proteins (Abidi et al., 2023). Conjugating the nanoparticles for diagnostic purposes will improve sensitivity and specificity that can lead to early detection methods. This will help in facilitating timely intervention when treatment options are to be explored. In some cases, quantum dots, which are basically semiconductor nanocrystals, are used as they are highly versatile labels in imaging applications. They can be designed in such a way to emit specific wavelengths of light when excited, which can be made use of to visualize neuronal changes at the nanoscale, thereby locating the abnormalities even before the clinical symptoms are visible.

### 3.1 Nanoparticles in neuro-inflammation and neuro-toxicity

The diagnosis and treatment of neurodegenerative diseases are centred around the CNS, and the treatment outcomes are critically marred by neuroinflammation and neurotoxicity. In PD, chronic neuroinflammation is triggered by the accumulation of misfolded α-synuclein along with mitochondrial dysfunction. Neuroinflammation is commonly known to exacerbate dopaminergic neuronal degeneration. It is observed that certain nanoparticles can either aggravate or alleviate this inflammatory cascade based on their size, surface charge, and composition. Selenium nanoparticles exhibit neuroprotective effects by mitigating oxidative stress, primarily through scavenging ROS, inhibiting α-synuclein aggregation, and modulating microglial activation. It is observed that these mechanisms reduce neuroinflammatory stress in PD models (Umapathy et al., 2025). However, despite their protective role in neuroinflammatory distress, nanoparticles are known to induce neurotoxicity. Nanoparticles are capable of disrupting neuronal membranes and activating pro-inflammatory pathways, leading to oxidative stress and apoptosis. Gold nanoparticles offered dose-dependent effects, with low concentrations reducing inflammation and higher doses triggering mitochondrial damage and glial cell activation (Kumar et al., 2022). Sometimes, biocompatibility and surface functionalization are integral to minimize the unintended neurotoxicity. Also, in cases where the BBB is compromised (as often observed in PD), the nanoparticles may end up in heaps around the substantia nigra region and this makes precision targeting and controlled release a high priority event to avoid exacerbating neurodegeneration (Cheng et al., 2022). However, in spite of this, there are nanoparticles that are capable of countering neuroinflammation without inducing toxicity. Non-invasive stimulation of brain regions by laser-activated nanoparticles is a better alternative approach to brain stimulation with cognitive side effects. By minimal systemic exposure, these nanoparticles can influence neuronal activity and motor symptoms in PD.

### 3.2 Nanoparticles in neuroprotection

Nanotechnology often offers a novel approach to developing therapeutic strategies aimed at preserving neural function and promoting recovery. By exploiting the distinct properties of nanomaterials, researchers are developing innovative therapeutic strategies to address the complex challenges of NDs. Current research in this area continues to refine these applications for exploring new nanomaterials that could be used for future neuroprotective interventions. Some of the notable examples related to the application of nanoparticles in the research related to various aspects of neurodegenerative diseases and the subsequent utilization for treatment are discussed here.

#### 3.2.1 Platinum nanoparticles

Platinum nanoparticles (PtNPs) are being researched extensively for their potential to mitigate neurotoxicity and oxidative stress in neurological conditions. Their unique physicochemical properties, such as high surface area and catalytic activity, provide them with a capacity to scavenge ROS, reduce inflammation, and protect neuronal cells from damage that are caused by NDs, toxins, or ischemic injury. The research revealed details on how PtNPs influence brain damage, cerebral blood flow (CBF), and oxidative stress in rats subjected to ischemic conditions. Also, despite their extremely low accumulation in the brain, these nanoparticles demonstrated significant neuroprotective properties (Filippov et al., 2023). It was found that PtNPs weakened post-ischemic hypoperfusion, attenuated neuronal apoptosis with minimal cell death in the hippocampus region, and improved glutathione redox status by preserving the glutathione redox balance and reduced oxidative damage, thus modulating oxidative stress also. However, they have extremely low bioavailability as it was shown that despite their neuroprotective effects, they were found in very low concentrations in brain tissue. Such effects were mediated through indirect mechanisms and not direct accumulation in brain tissue, proving PtNPs to be a significant candidate in the quest for effective neuroprotective agents. The study reported that PtNPs could activate neuroprotective signaling pathways, including the PI3K/Akt pathway, promoting neuronal survival and reducing apoptosis in neuronal cultures exposed to ischemic conditions. This suggests a promising avenue for therapeutic use in stroke and other ischemic brain injuries. Another study explored the neuroprotective potential of PtNPs that were synthesized from *Biophytum reinwardtii* in a zebrafish model of PD (Bandaru et al., 2024a). The disease was induced by MPTP and the zebrafish were treated with different concentrations of these nanoparticles. The effects on locomotor activity, oxidative stress markers, and catecholamine levels were assessed and the results showed that PtNPs significantly improved movement, increased antioxidant levels, and restored catecholamine balance, suggesting their potential for neuroprotection in PD. These findings highlight the potential of platinum nanoparticles in treating acute cerebrovascular disorders through systemic antioxidant and neuroprotective pathways. Given their unique properties to bring about antioxidant, anti-inflammatory, and electrochemical changes, PtNPs are gaining much traction in neurodegenerative disease research as well. They can act as nanozymes and ROS scavenging molecules in neurovascular units, where even as 5 nm sized particles, they can act as antioxidant enzyme-mimics that can efficiently mime the natural enzymes like catalase and superoxide dismutase. The PtNPs scavenged ROS in neurovascular cellular models, effectively protecting neurons and glial cells from oxidative damage. The impetus behind neurodegenerative cascades in conditions such as Alzheimer’s and Parkinson’s often stems from oxidative damage to vulnerable neural and glial cells (Tarricone et al., 2023). It was observed that upon administration of PtNPs, the catalytic properties of the nanozyme activated the lysosomal environment, turning it into tiny, intracellular ‘microreactors’ that lowered mitochondrial stress, thus preserving the BBB integrity. When tested under kainic acid-induced excitotoxic conditions that are a proxy for neurodegeneration in an *ex vivo* rat hippocampal model, PtNPs showed no particular impairment of neuronal viability in CA1 and CA3 regions (parts of hippocampus), even under pathological stress. However, it was observed that they triggered microglial activation, clearly proposing an immune-modulatory role. This points to the possibility of harnessing PtNPs for therapeutic applications with controlled dosing (Gulino et al., 2021). Another study in a rat model showed that electrodes coated with PtNP (used for deep brain stimulation) displayed reduced and stabilized impedance over 4 weeks of stimulation. This result points to the possibility of enhanced precision and longevity of neurostimulation therapies for Parkinson’s and other movement disorders, indicating PtNPs’ utility in neuroelectronic interfaces (Angelov et al., 2024).

#### 3.2.2 Titanium dioxide nanoparticles

Titanium dioxide nanoparticles (TiO_2_ NPs) are widely used in consumer products. However, they are being explored in therapeutic uses as well. Even though the neurotoxic effects of TiO_2_ have raised concerns due to their environmental presence (Aschner et al., 2023) and human exposure, it is highlighted that TiO_2_, when in nanoform, can cross the BBB. The exact mechanism of how TiO_2_ NPs cross the BBB is still unclear, but it is understood that once in the brain, it can induce oxidative stress and neuroinflammation, leading to disruption in brain biochemistry, followed by neuronal damage. This may influence behavioural disorders and neurodegenerative diseases, including PD. The neurotoxic potential of TiO_2_ NPs is influenced by the physicochemical properties and how they are exposed to the site of action during disease conditions. Another study investigated the neurotoxic effects of TiO_2_ NPs on neuronal, PC-12 cells. The study mainly focused on cytotoxicity, dopaminergic gene expression, and acetylcholinesterase inhibition. When two differently sized nanoparticles (10 nm and 22 nm) along with polyvinylpyrrolidone (PVP)-coated TiO_2_ NPs were used for the study, it was observed that at concentrations ≥10 μg/mL, it induced dose-dependent cytotoxicity due to increased ROS and nitrogen species (Suthar et al., 2023). Elevated inflammatory markers (like IL-6 and TNF-α), mitochondrial dysfunction, and apoptosis-related caspase-3 activation were observed, accompanied by acetylcholinesterase inhibition. These results suggest a potential neurochemical disruption in the brain. However, dopaminergic gene expression remained unaffected at lower exposure levels, which was considered an interesting observation. The surface coating of the nanoparticles with PVP reduced neurotoxic effects, highlighting its potential for safer medical applications. *In vitro* studies using neuronal and glial cell lines revealed that TiO_2_ NPs can induce neuronal cell damage through the induction of oxidative damage via the generation of ROS. It was also found that the cells encounter reduced ATP levels invoked by mitochondrial dysfunction. An exceptionally high dose or prolonged exposure to TiO_2_ NPs has been shown to trigger apoptosis/necrosis (Song et al., 2015). Further, specific inflammatory responses were observed with microglial activation and increased expression of pro-inflammatory cytokines (like IL-6, TNF-α), specifying role for TiO_2_ NPs in neuroinflammation - a key driver in diseases like Alzheimer’s and Parkinson’s. When applied to *in vivo* rodent models via inhalation or intravenous routes, the TiO_2_ NPs were found to be accumulated in various regions of the brain, including the hippocampus and cortex. Behavioral assays were conducted in the test organism, which revealed specific impairment of spatial memory and learning, consequently correlating with histopathological changes in brain tissue. Evidence of BBB disruption was observed, further facilitating the entry of other neurotoxicants. Assays on neurodevelopmental impact through prenatal exposure in these models showed altered brain development, pointing to potential risks during gestational periods (Zhang, Song et al., 2023).

#### 3.2.3 Gold nanoparticles

The neuroprotective effects of gold nanoparticles (AuNPs) have been extensively studied for the treatment of PD. The nanoparticles were found to inhibit the generation of ROS and modulate the expression of pro-apoptotic and anti-apoptotic proteins (Piktel et al., 2021), suggesting that AuNPs have the potential to serve as a therapeutic agent in neurodegenerative diseases, including PD (de Bem Silveira et al., 2021). AuNPs are known to mitigate neuronal apoptosis that is caused by glutamate toxicity, which is considered a key factor in neurodegenerative diseases. Glutamate is an excitatory neurotransmitter, and its presence can lead to excessive calcium influx triggering oxidative stress, mitochondrial dysfunction, and caspase activation, ultimately resulting in neuronal cell death. It was inferred from the study that AuNPs reduced oxidative stress by scavenging ROS, and stabilized mitochondrial function by preventing the release of cytochrome c. It was found to inhibit the activation of caspase-3, strengthening its role in induction of apoptosis. It was also found to modulate neuroinflammatory pathways by reducing inflammatory cytokines. All these protective roles highlight the potential of AuNPs as therapeutic agents for neurodegenerative conditions like AD and PD (de Bem Silveira et al., 2021; Chiang et al., 2024). Further, the potential of AuNPs in diagnosing and treating neurodegenerative diseases were studied. It was understood that AuNPs could be functionalized to explicitly target the key pathological markers of neurodegenerative diseases - Tau and α-synuclein proteins. AuNPs enabled the detection of these proteins even at low concentrations in biological samples like blood or cerebrospinal fluid, enabling early and accurate diagnosis of the disease. AuNPs can also serve as drug delivery platforms, enabling the transport of therapeutic agents directly into the brain, thus improving treatment precision while maintaining minimal side effects (Tapia-Arellano et al., 2024). However, while the use of AuNPs looks promising, safety and biocompatibility are major concerns, requiring rigorous preclinical and clinical studies before widespread medical use. *In vitro* assays revealed the neuroprotective effect of AuNPs through anti-inflammatory activity, where supplementing the cultured microglial cells with AuNPs reduced neuroinflammation, as evidenced by the downregulation of pro-inflammatory cytokines like TNF-α and IL-6. These are generally elevated in AD and PD pathology (Chiang et al., 2024). Targeting of Tau and α-synuclein by functionalized AuNPs revealed a selective binding of the NPs to misfolded proteins (Tau in AD and α-synuclein in PD), thereby inhibiting their aggregation and toxicity in the tested neuronal cells (Tapia-Arellano, 2024). Treatment with AuNPs also revealed the antioxidant properties, where ROS was scavenged to protect the neurons from oxidative damage - a key driver in neurodegeneration. *In vivo* studies in mouse models showed that when conjugated with therapeutic agents, AuNPs improved cognitive function with reduced amyloid plaque burden. Drug delivery across the BBB was also observed to increase bioavailability in the brain. When targeted towards α-synuclein aggregates, AuNPs reduced motor deficits and neuroinflammation in PD animal models. This ability of AuNPs to deliver neuroprotective compounds directly to the regions of the brain that are affected by PD or other neurodegenerative conditions revealed improved therapeutic outcomes. AuNPs have even been helpful to mitigate ischemic damage after stroke – they act by reducing oxidative stress and inflammation, thereby preserving the integrity of neuronal cells, providing cerebrovascular protection (Chiang et al., 2024).

#### 3.2.4 Selenium nanoparticles

Oxidative stress followed by dopaminergic neuron damage and protein misfolding are considered as major contributors to neurodegeneration. The neuroprotective potential of selenium nanoparticles (SeNPs) in treating PD was explored (Umapathy et al., 2024; Kalčec et al., 2023). SeNPs are found to act as antioxidants, scavenging the ROS and reducing oxidative damage. They also help in maintaining mitochondrial integrity by preventing cytochrome c release and apoptosis, which are crucial for neuronal survival. SeNPs are known to cross the BBB as any other nanoparticles and therefore, targeted delivery of SeNPs to affected regions of the brain are possible. SeNPs also suppress neuroinflammation by lowering the levels of pro-inflammatory cytokines like IL-6 and TNF-α, which significantly contribute to PD progression. The accumulation of misfolded proteins like α-synuclein and tau are inhibited by SeNPs. All these results point to the fact that SeNPs could be used as a potential therapeutic agent in neurodegenerative diseases like Parkinson’s (Umapathy et al., 2024).

Another research also discussed the potential of SeNPs for use as a mode of delivery for transporting drugs to treat PD. Since PD is characterized by dopaminergic neuron degeneration, the mode of action of SeNPs in enhancing drug transport across BBB was investigated and the therapeutic efficacy was also examined. It was understood that when SeNPs were functionalized with PVP and polysorbate 20 (Tween), it improved stability and drug-binding capacity. Also, L-DOPA and dopamine were successfully loaded onto SeNPs, showing that they have strong binding interactions that could enhance drug delivery. SeNPs also improved BBB permeability, allowing better drug absorption in brain cells, which is important in treatment of diseases like PD. Further, *in vitro* studies demonstrated efficient drug uptake by human brain endothelial cells (Kalčec et al., 2023). The antioxidant, anti-inflammatory, and neuroprotective properties of SeNP in both *in vitro* and *in vivo* studies provided compelling evidence of their therapeutic potential, especially in neurodegenerative disease conditions like AD, PD, and amyotrophic lateral sclerosis (ALS). Assays with the *in vitro* neuronal cell cultures revealed that the treatment with SeNPs reduced β-amyloid aggregation and inhibited tau hyperphosphorylation, which are the hallmarks of neurodegenerative diseases. The modulation of neuroinflammation through the downregulation of pro-inflammatory cytokines (e.g., IL-1β, TNF-α) in microglial cells indicated the ability of SeNPs to suppress neuroinflammatory cascades (Vicente-Zurdo et al., 2024). *In vivo* evidence in AD mouse models (transgenic AD mice treated with SeNP) revealed improved cognitive performance mediated by behavioral tests. It showed reduced amyloid plaque burden in the hippocampus, and enhanced antioxidant enzyme activity through glutathione peroxidase activity. When administered in PD-induced rodents, SeNPs showed protection of dopaminergic neurons in the substantia nigra with improved motor coordination and reduced tremors. They also lowered neuroinflammation markers, suggesting disease-modifying potential for the SeNPs. Further, functionalized SeNPs demonstrated the ability to cross the BBB, thereby delivering the therapeutic payloads directly to affected regions of the brain (Rajeshkumar et al., 2019).

#### 3.2.5 Magnetite nanoparticles

The neuroprotective effects of magnetite nanoparticles (Fe_3_O_4_ NPs) were explored and it was found that they exhibited significant ability to reduce oxidative stress (Ucar et al., 2022), modulate neuroinflammation (Tamjid et al., 2023), and support mitochondrial function (Zhang et al., 2021b). These abilities of magnetite nanoparticles help the brain to preserve dopaminergic neurons in neurodegenerative diseases like Parkinson’s and Alzheimer’s. The potential of magnetite nanoparticles to act as drug-delivery systems was reviewed and it was discerned that the magnetite nanoparticles served as excellent agents for targeted therapy, improving treatment efficiency while minimizing systemic side effects. Research on the neuroprotective potential of Fe_3_O_4_ NPs in a PD model, focused on their ability to enhance neuronal survival, and improve motor function. Improved mitochondrial functions were observed, leading to enhanced energy metabolism and reduced apoptosis. They were found to modulate neuroinflammatory pathways by lowering levels of pro-inflammatory cytokines like IL-6 and TNF-α. Enhanced drug delivery potential suggested that Fe_3_O_4_ NPs could be used as carriers for targeted PD therapy (Khadrawy et al., 2024). Another study investigated the neuroprotective potential of iron oxide nanoparticles (IONPs) synthesized through green-route via ascorbic acid (AA-IONPs) in PD using both *in vitro* and *in vivo* models. The AA-IONPs were found to reduce neuroinflammation by lowering the levels of nitric oxide, prostaglandin E2, IL-6, and IL-1 in murine microglial BV2 cells. *In vivo* experiments on PD-induced C57BL/6 mice demonstrated enhanced motor coordination and reduced neuroinflammation after AA-IONP treatment. These findings suggest that AA-IONPs could serve as an effective nano-drug for neuroprotection through their anti-inflammatory and antioxidant properties (Li et al., 2023). The neuroprotective effects of IONPs in a PD model were studied (Khadrawy et al., 2024). The findings indicated that these nanoparticles could effectively deliver curcumin and enhance its bioavailability in neuronal cells. They not only demonstrated antioxidant activity but also modulated neuroinflammation by reducing the levels of inflammatory cytokines, providing a dual mechanism of action. In AD imaging, Fe_3_O_4_ NPs have been used as MRI contrast agents for the early detection of AD (Ulanova et al., 2020). The magnetic properties of Fe_3_O_4_ NPs enhance the resolution of the images to such an extent that when functionalized with imaging molecules, they can target amyloid plaques. Neuroprotective signaling in rodent models showed that Fe_3_O_4_ NPs activated the neuroprotective pathways and supported mitochondrial function, thereby reducing apoptosis and maintaining neuronal viability (Abdelmonem, et al., 2024).

#### 3.2.6 Silica nanoparticles

Silica nanoparticles (SiNPs) have been studied for their ability to deliver therapeutic agents across the BBB (Bhatane et al., 2023). The potential of functionalized SiNPs as drug-delivery systems for neuroprotective treatments was examined and it was found that they enhanced the cognitive function in neurodegenerative diseases like PD. When SiNPs were functionalized with targeting ligands, they improved the BBB penetration and it brought about precise drug delivery to neuronal tissues. When neuroprotective drugs were loaded onto SiNPs, it also ensured sustained therapeutic effects through enhanced stability and bioavailability. Further, *in vitro* and *in vivo* studies with SiNPs demonstrated reduced oxidative stress, improved neuronal survival, and enhanced cognitive function in disease models. They also minimized systemic toxicity, making them a promising biocompatible nanocarrier for neurological treatments. Since mesoporous SiNPs offer high surface area and tunable pore size, they are ideal for targeted drug delivery. They protect encapsulated drugs from premature metabolism, ensuring controlled release and stability. They are further explored for treating brain tumors, epilepsy, depression, and multiple sclerosis as their biocompatibility and surface conjugation enhance therapeutic efficacy while minimizing side effects (Bhatane et al., 2023). When used as nanocarrier system, they showed high biocompatibility and low toxicity, indicating its potential use in neuroprotection strategies. *In vitro* studies on SH-SY5Y (human neuroblastoma) cell model revealed that exposure to SiNPs induced excessive ROS generation, Ca^2+^ overload, and mitochondrial dysfunction. They were shown to activate both parthanatos and caspase-dependent apoptotic pathways, subsequently leading to neuronal cell death (Ma et al., 2025). However, when the cells were treated with Olaparib, a PARP inhibitor and Z-VAD, a caspase inhibitor, these observed effects were significantly mitigated, suggesting a potential for controlled therapeutic modulation. Further, functionalized SiNPs were shown to promote α-synuclein aggregation in dopaminergic neurons, thus mimicking Parkinson’s pathology. This highlights both the diagnostic potential (by modelling disease mechanisms) and the need for surface modification strategies to reduce neurotoxicity. In A53T transgenic mouse model, the intranasal administration of SiNPs led to the hyperphosphorylation and aggregation of α-synuclein, mitochondrial impairment, oxidative stress, dysfunction of autophagy and neuronal apoptosis. This is a clear indication of the penetrative effect of SiNPs on the CNS, resulting in potential for targeted therapeutic delivery in PD-like pathological conditions. Neurodevelopmental toxicity studies showed that prenatal exposure to SiNPs altered brain development in rodent models, emphasizing the importance of dose control and timing in therapeutic applications (Yang et al., 2022; Ma et al., 2025).

#### 3.2.7 Graphene and graphene oxide

The neuroprotective potential of nano-graphene oxide (NGO) in combating PD was tested by targeting ROS scavenging and anti-inflammatory mechanisms. It was found that NGOs affected SH-SY5Y cells and a 6-hydroxydopamine (6-OHDA)-induced Parkinsonian rat model - it protected the tested cells against 6-OHDA-induced toxicity by reducing oxidative stress. It also suppressed microglial activation, which is linked to neuroinflammation in PD. In the rat models tested, NGO was found to have improved akinesia symptoms and reduced contralateral rotations in response to apomorphine, which implied neuroprotection through increased tyrosine hydroxylase-positive cells in NGO-treated rats, suggesting preservation of dopaminergic neurons. Overall, the study presented NGO as a promising candidate for PD treatment (Kim et al., 2023a). While discussing the neuroprotective effects of graphene-based nanoparticles, it was apparent that they can influence many molecular events that contribute to PD neurodegeneration like autophagy, inflammation, and oxidative stress. They are explored for their role in interventions related to nanomedicine and regenerative approaches to restore neuronal function and was believed to improve early-stage PD detection through graphene-based biosensors (Oz et al., 2023). When exploring the therapeutic potential of graphene nanoparticles, a study was conducted to check how graphene oxide (GO) nanoflakes could target dysfunctional synaptic plasticity in the amygdala, particularly in conditions like post-traumatic stress disorder (PTSD). By selectively targeting glutamatergic synapses, it was found that GO nanoflakes with small lateral dimensions (s-GO) could transiently interact with glutamatergic synapses in the hippocampus, reducing neurotransmitter release. This was of specific significance as PTSD is linked to hyperactive glutamatergic transmission in the lateral amygdala (LA). The study further investigated whether s-GO can modulate this excessive activity to restore normal synaptic function and examined the subcellular targets of s-GO and its interaction with potentiated synapses, potentially reducing long-term potentiation (LTP) associated with PTSD. The study also explored s-GO as a nanocarrier for drug delivery, using neuropeptide Y (NPY) as a biologically active molecule to enhance therapeutic effects (Pati, 2022). The promising role of graphene oxide nanoparticles in neuroprotection were highlighted and their ability to promote neuronal differentiation makes it a compelling candidate for regenerative therapies in neurodegenerative diseases.

#### 3.2.8 Silver nanoparticles

The antioxidant, anti-inflammatory, and neuroprotective properties of silver nanoparticles (AgNPs) have made them a promising candidate in neurodegenerative disease control. A particular study highlighted the neuroprotective potential of AgNPs synthesized using a red pigment from *Streptomyces* sp. A23, isolated from Algerian bee pollen on SH-SY5Y cells and their potential therapeutic applications (Mohamed et al., 2025). The sample was tested at concentrations of 2, 4, and 8 μg/mL, showing cytotoxic effects on the experimental cells. Significant neuroprotective activity of AgNPs was evident even at lower concentrations (1 mM, pH 7), where a dose-dependent effect was noted. The study also indicated that AgNPs induced their neuroprotective activity through mitigation of oxidative stress, which is a major contributor to the progression of neurodegenerative diseases. These findings suggested that AgNPs synthesized using *Streptomyces* sp. A23 could be used as a promising neuroprotective agent. However, the authors are of the opinion that it warrants further investigation for use as a drug for PD, AD, and other neurodegenerative disorders. AgNPs are known to cause significant neuronal death. This has raised serious concerns about their long-term safety. Research suggests that AgNPs can cross the BBB and accumulate in the brain, leading to neurotoxicity and neuronal degeneration (Janzadeh et al., 2022). While AgNPs hold potential for neuroprotection, their toxicity risks must also be carefully assessed and managed to ensure safe and effective applications in neurodegenerative disease treatment. Since neurodegenerative diseases like Parkinson’s already involves progressive neuronal loss, AgNP-induced neuronal death could worsen disease progression rather than help. AgNPs are also found to be linked to microglial activation, which may exacerbate neuroinflammation. All these indicate that AgNPs can damage synaptic structures, potentially impairing cognitive and motor functions. Such research underscores the potential dangers of AgNP exposure, urging caution in their biomedical applications.

#### 3.2.9 Chitosan nanoparticles

Nanoparticles derived from chitosan, a biodegradable and biocompatible polysaccharide obtained from chitin (which is found in the exoskeletons of crustaceans) are called chitosan nanoparticles (CsNPs). These types of nanoparticles have gained significant attention in neuroscience, particularly in addressing neurodegenerative diseases. They have unique physicochemical properties, like high permeability across biological barriers, low toxicity, and inherent antioxidant and anti-inflammatory effects which make them promising candidates for drug delivery systems in neurodegeneration. The neuroprotective potential of CsNPs as carriers for *Ginkgo biloba* extract (GBE), specifically targeting oxidative stress-induced damage in SH-SY5Y cells were examined (Karavelioglu and Cakir-Koc, 2021) and it was understood that these nanoparticles enhanced neuroprotection through improved bioavailability and increasing cell viability from 60% to 92.3%. It was revealed that GBE-loaded CsNPs effectively scavenged ROS, thereby reducing oxidative damage in neuronal cells. The ionic gelation method used for CsNP synthesis resulted in high encapsulation efficiency (97.4%) and ensured controlled release and better cellular uptake. These findings suggested that GBE-CsNPs could be developed as food supplements or therapeutic agents for conditions like AD and PD. Further, studies have shown the neuroprotective potential of CsNPs, improving outcomes in animal models of neurodegeneration and neuroinflammation. The potential of CsNPs as a therapeutic channel for neurological disorders were explored, and the ease of functionalization make these nanoparticles promising candidates for drug delivery in neurodegenerative diseases like Alzheimer’s, Parkinson’s, epilepsy, migraine, psychotic disorders, and brain tumors (Vahab et al., 2024). CsNPs help overcome the BBB by enhancing drug absorption, protecting drugs from degradation, and enabling targeted delivery. Additionally, it was observed that surface modifications of CsNPs allow for the attachment of specific ligands or molecules, improving precision in drug delivery to neuronal cells. However, despite these advancements, challenges remain in large-scale production, regulatory approvals, and long-term safety issues that need to be addressed for the future use of CsNP-based therapies in neurological disorders.

#### 3.2.10 Polymeric nanoparticles

By encapsulating multiple neuroprotective agents, polymeric nanoparticles can modulate immune responses and even promote neuronal regeneration by providing multifunctional therapeutic benefits. By functionalizing with specific ligands, these nanoparticles can improve targeted drug delivery to damaged neurons. The polymeric nanoparticle-based therapies offer a revolutionary shift in precision medicine, with better drug absorption, enhanced cellular targeting, and potentially reduced side effects. Many researchers use polymeric nanoparticles to encapsulate neuroprotective compounds and deliver them to the site of action. For example, the stability and bioavailability of curcumin, a natural compound was significantly improved when encapsulated in a polymeric substance. The specially formulated, curcumin-loaded nanoparticles significantly reduced amyloid-beta plaque accumulation and inflammation, showcasing the potential of nanotechnology to enhance the therapeutic effects of existing compounds (Pei et al., 2024). Another study explored the neuroprotective potential of edaravone-loaded mPEG-b-PLGA polymeric nanoparticles in an *in vitro* ischemia model using the SH-SY5Y cell line. It was observed that the polymeric encapsulation improved edaravone’s bioavailability and stability and considerably lowered the activities of ROS and nitric oxide (NO), which are major contributors to neuronal damage. The expression of pro-apoptotic gene Bax was downregulated while the anti-apoptotic genes HSP70 and Bcl-2 were upregulated, promoting cell survival. Improved neuroprotection was observed where the cells treated with edaravone-loaded nanoparticles showed better resistance to ischemia-induced damage compared to free edaravone (EDV). By enhancing edaravone’s therapeutic effects, these nanoparticles could pave the way for more effective neuroprotective strategies in stroke and other neurodegenerative conditions (Sharifyrad et al., 2022).

Spinal cord injury (SCI) leads to progressive neuronal damage, driven mainly by oxidative stress and inflammation. In such cases, the ROS-scavenging were used for neuroprotection to mitigate oxidative stress and inflammation (Zhang et al., 2021a). These nanoparticles offer a promising therapeutic strategy by reducing secondary injury, potentially improving recovery outcomes. The lipid nanoparticles effectively neutralized harmful free radicals, reducing oxidative damage in injured spinal cord tissue. They also suppressed the secretion of pro-inflammatory cytokines (such as IL-1β, TNF-α, and IL-6), helping to control inflammation. Targeted accumulation of the nanoparticles at the injury site enhanced their therapeutic effects. Functional recovery was observed in animal models, where treatment with these nanoparticles led to better axonal protection and improved motor function.

#### 3.2.11 Lipid nanoparticles

RNA therapies, including messenger RNA (mRNA) and siRNA, offer promising approaches for gene silencing and protein expression in neurodegenerative diseases. The mRNA, siRNA and antisense oligonucleotides (ASOs) can correct genetic defects, silence harmful genes, or restore normal protein function (Lee et al., 2022). A cutting-edge study concentrated on lipid-based nanoparticles that served as efficient RNA carriers, protecting RNA from degradation and enhancing its delivery across the BBB. Various types of lipid-based platforms include liposomes, lipoplexes, solid lipid nanoparticles (SLNPs), lipid nanoparticles (LNPs), nanoemulsions (NEs), nanoliposomes, nanophytosomes, and nanostructured lipid carriers (NLCs), each with unique properties for targeting the CNS (Tsakiri et al., 2022). Surface modifications of LNPs improve target specificity, allowing RNA therapies to reach affected neurons more effectively. This strategy is based on LNPs as carriers for RNA-based therapeutics in neuroprotection. The delivery of siRNA targeting neurotoxic pathways in models of amyloid-beta toxicity was studied and the results indicated that LNPs improved the uptake of siRNA by neuronal cells, leading to a significant reduction in neurotoxic effects and suggesting potential therapeutic applications in neurodegenerative diseases. Thus, lipid-based nanoparticles are emerging as a powerful tool for treating neurodegenerative diseases by enhancing drug delivery, reducing toxicity, and improving therapeutic efficacy (Fernandes et al., 2021).

#### 3.2.12 Hybrid nanoparticles combining different materials

Hybrid nanoparticles that integrate different materials like polymers, lipids, metals, and biocompatible nanomaterials are emerging as a promising approach by offering enhanced therapeutic capabilities for treating neurological disorders. These hybrid systems can leverage the unique properties of each component to optimize drug delivery, whereby they can reduce toxicity, and improve brain targeting. Their additional ability to cross the BBB and deliver antioxidants, anti-inflammatory agents, and neurotrophic factors make them valuable candidates for mitigating neurodegeneration in most of the neurodegenerative diseases, such as Alzheimer’s, Parkinson’s, and stroke-induced brain injury. Further, the multifunctionality of hybrid nanoparticles allows for controlled drug release, improved cellular uptake, and enhanced neuroprotective effects, making them superior to its conventional counterpart, namely, single-material nanocarriers (Kumar and Kumar 2024). Many of the research with hybrid nanoparticles have shown that combining lipid-based carriers with polymeric shells or metallic cores can significantly boost drug stability, bioavailability, and therapeutic efficacy. These advanced systems not only protect neurons from oxidative stress and inflammation but also promote neuronal regeneration, offering new possibilities for treating CNS disorders. While investigating the therapeutic potential of a polymer/lipid hybrid nanoparticle (PLHNPs) loaded with quetiapine fumarate (QF) in a cuprizone-induced schizophrenia model in mice, the drug delivery efficiency and behavioral and neurological outcomes in schizophrenia were revealed. Further, the hybrid nanoparticles demonstrated high entrapment efficiency (99.68%) and a sustained release profile, with 95% of QF released over 28 days. Moreover, mice treated with the optimized hydrogel-based formulation (HF-G3) showed significant recovery from schizophrenia-like symptoms, compared to untreated cuprizone-fed mice. This was a significant study that revealed reduced levels of pro-inflammatory cytokines (TNF-α, IL-1β), gamma-aminobutyric acid (GABA), and glial fibrillary acidic protein (GFAP), indicating reduced neuroinflammation and improved neuronal integrity of the formulation (Elkasabgy et al., 2023). In addition to this, the study ensured that intramuscular administration prolonged therapeutic effects, reducing the need for frequent dosing. Generally, quetiapine fumarate has very low oral bioavailability (9%) and the study successfully demonstrated the potential of PLHNPs in sustained drug delivery, even in case of drugs with very low bioavailability.

Auranofin is an orally administered gold compound primarily used to treat rheumatoid arthritis, but recently, it has been studied for neuroprotective effects, particularly in PD models, where it helps reduce oxidative stress and neuroinflammation (Soni et al., 2025). The ability of auranofin-loaded chitosan-lipid hybrid nanoparticles (AUF-CLHNPs) to modulate GSK-3β/Nrf2/HO-1 signaling pathways in a rotenone-induced PD model was studied and it was found that AUF-CLHNPs significantly improved auranofin’s brain penetration, overcoming its limited natural bioavailability. The hybrid nanoparticles were found to restore motor function by reducing oxidative stress, and protecting the dopaminergic neurons in the substantia nigra, a key brain region affected in PD. The anti-inflammatory responses were influenced where UF-CLHNPs phosphorylated GSK-3β, leading to upregulation of Nrf2/HO-1, affecting oxidative pathway as well. The pro-inflammatory cytokine levels (TNF-α, IL-1β) were downregulated and neuronal integrity was restored. Thus, the study successfully demonstrated the use of a novel drug delivery approach that enhanced auranofin’s therapeutic efficacy in PD. The hybrid nanoparticle formulation ensures sustained drug release, improving treatment outcomes.

#### 3.2.13 2-D materials

**FIGURE 5:**
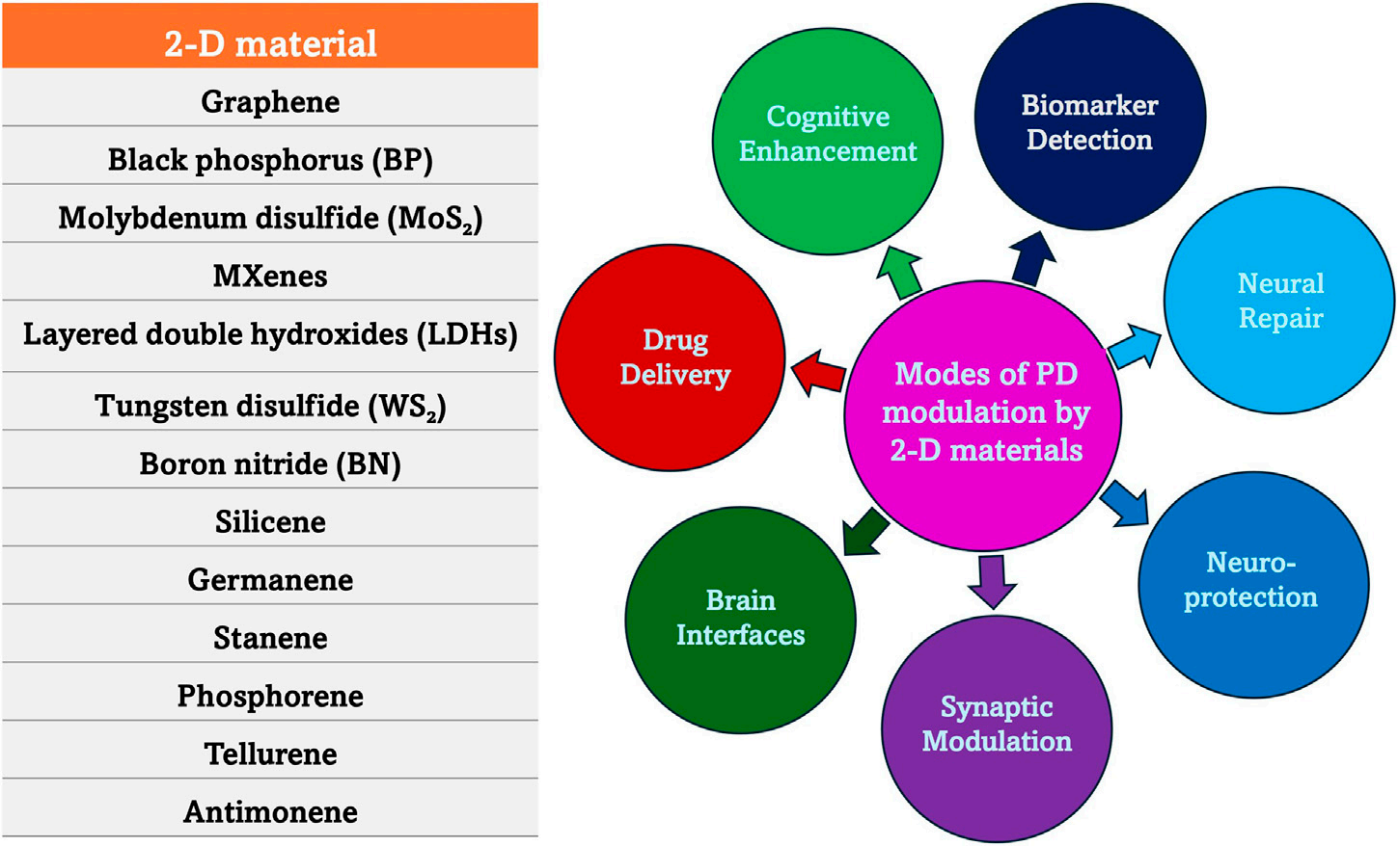
Mode of action of 2-D materials in PD. 2-D materials exhibit unique physicochemical properties, including high surface area, biocompatibility, and tunable electronic characteristics, making them promising candidates for therapeutic applications. Their ability to regulate oxidative stress, neuroinflammation, mitochondrial function, and α-synuclein aggregation positions them as innovative tools in the fight against PD.

The exceptional physicochemical properties of two-dimensional (2-D) materials made them promising candidates for the diagnosis and treatment of neurodegenerative diseases and recently, they gained immense attention in biomedical research due to these properties. Some of these materials like graphene, transition metal dichalcogenides (TMDCs), molybdenum and black phosphorus, exhibit high surface area, excellent biocompatibility, and tunable electronic characteristics, which enhance their interactions with biological systems. In the field of diagnostics, 2-D materials can be integrated into biosensors for highly sensitive and selective detection of biomarkers associated with conditions like AD and PD (Wu et al., 2021). Additionally, their unique optical, electrical, and magnetic properties enable early disease detection through techniques such as fluorescence imaging, electrochemical sensing, and MRI enhancement. However, 2-D materials are of assistance beyond diagnosis as they are valuable in neuroprotective therapies as well. The targeted delivery of neuroprotective agents across the BBB is mainly attributed to their increased drug-loading capacity and controlled release mechanisms. This is based on the principle of increasing treatment efficacy while minimizing systemic side effects. The antioxidant and anti-inflammatory activities also affect the intrinsic neuroprotective properties, which help in mitigating oxidative damage and neuroinflammation that are considered as two main drivers of neurodegeneration (Lee et al., 2020). The ongoing advancements in the field of 2-D materials for neurodegenerative therapies hold great potential to revolutionize precision medicine as they can be more efficient, targeted, and less invasive than other conventional therapeutic strategies. For example, the therapeutic potential of the nano-bio interactions of 2-D molybdenum disulfide (MoS_2_), an ultrathin nanomaterial, is being explored. It is observed that 2-D MoS_2_ maintains high anisotropy, surface-to-volume ratio, chemical functionality, and mechanical strength which makes MoS_2_ a promising material for biomedical applications, including drug delivery, regenerative medicine, biosensing, and bioelectronics (Yadav et al., 2019; Jin et al., 2025). MoS_2_ interacts with biological systems, influencing the intracellular trafficking, biodistribution, and biodegradation of therapeutic drugs. MoS_2_ is also found to interact with proteins and specific cell types, such as immune cells and progenitor stem cells, which determine its short-term and long-term biocompatibility (Roy et al., 2022). Also, 2-D MoS_2_ belong to the TMDCs that can contribute to neuroprotection by mimicking biological synapses and neurons, enhancing neuronal plasticity and repair. Their high surface reactivity and tunable electronic properties allow them to interact with neural networks, supporting synaptic modulation and neuroregeneration. Research suggests that TMDC-based neuromorphic devices can help reduce oxidative stress and neuroinflammation, two major contributors to neurodegenerative diseases. By facilitating efficient signal transmission and memory retention, these materials may aid in cognitive restoration and neural repair (Ma et al., 2023). These TMDCs can be employed to make flexible neuromorphic devices that contribute to neuroprotection by mimicking biological synapses and neurons. These devices offer high adaptability and plasticity, making them promising for brain-machine interfaces and neural repair technologies (Ke et al., 2024; Ma et al., 2023). Some of the modes of PD modulation by 2-D materials are represented in Figure 5.

#### 3.2.14 Exosomes

**FIGURE 6:**
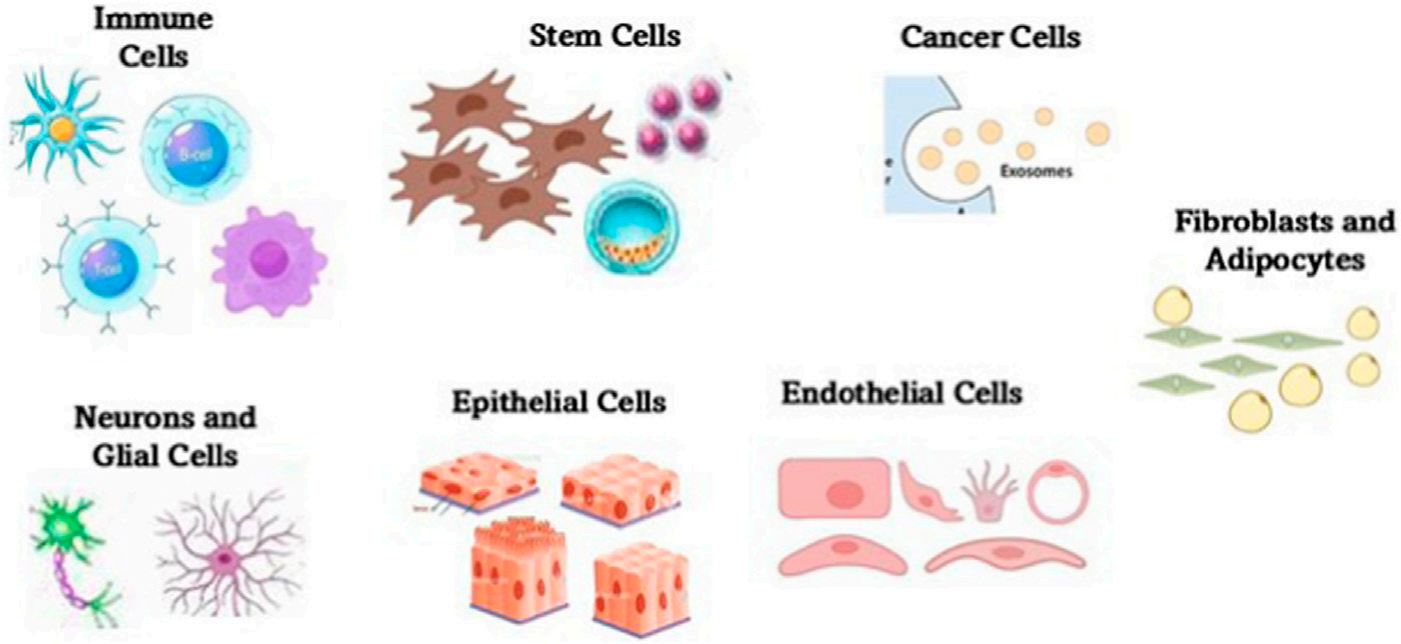
Major cellular sources of exosomes within the central nervous system. The sources include neurons, astrocytes, microglia, oligodendrocytes, and endothelial cells among others. Each cell type contributes distinct molecular cargo to exosomes, influencing processes such as neuroinflammation, synaptic signaling, and neuroprotection. The exosomes play a critical role in intercellular communication and are increasingly recognized for their diagnostic and therapeutic potential in neurodegenerative diseases.

**FIGURE 7:**
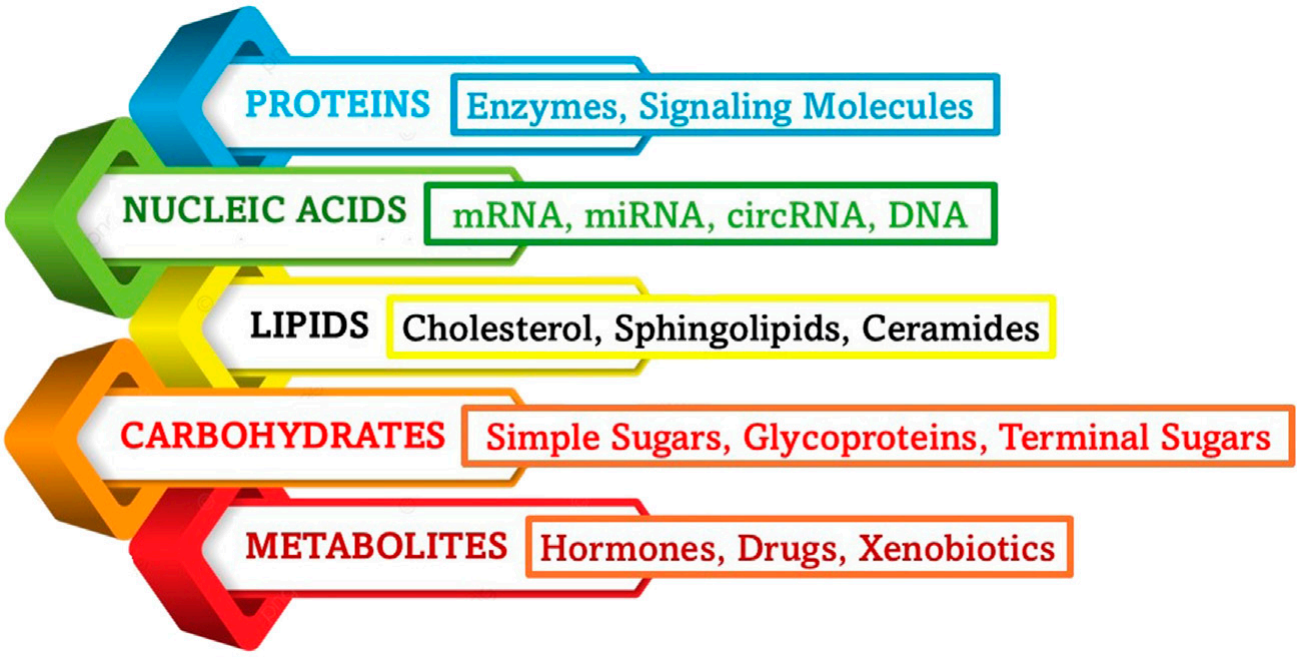
Main types of exosomal cargo. The bioactive molecules that act as cargo for exosomal delivery play key roles in intercellular communication, influencing processes such as inflammation, synaptic plasticity, and neuroprotection. The composition of exosomal cargo varies depending on the cell of origin and physiological or pathological conditions.

**FIGURE 8:**
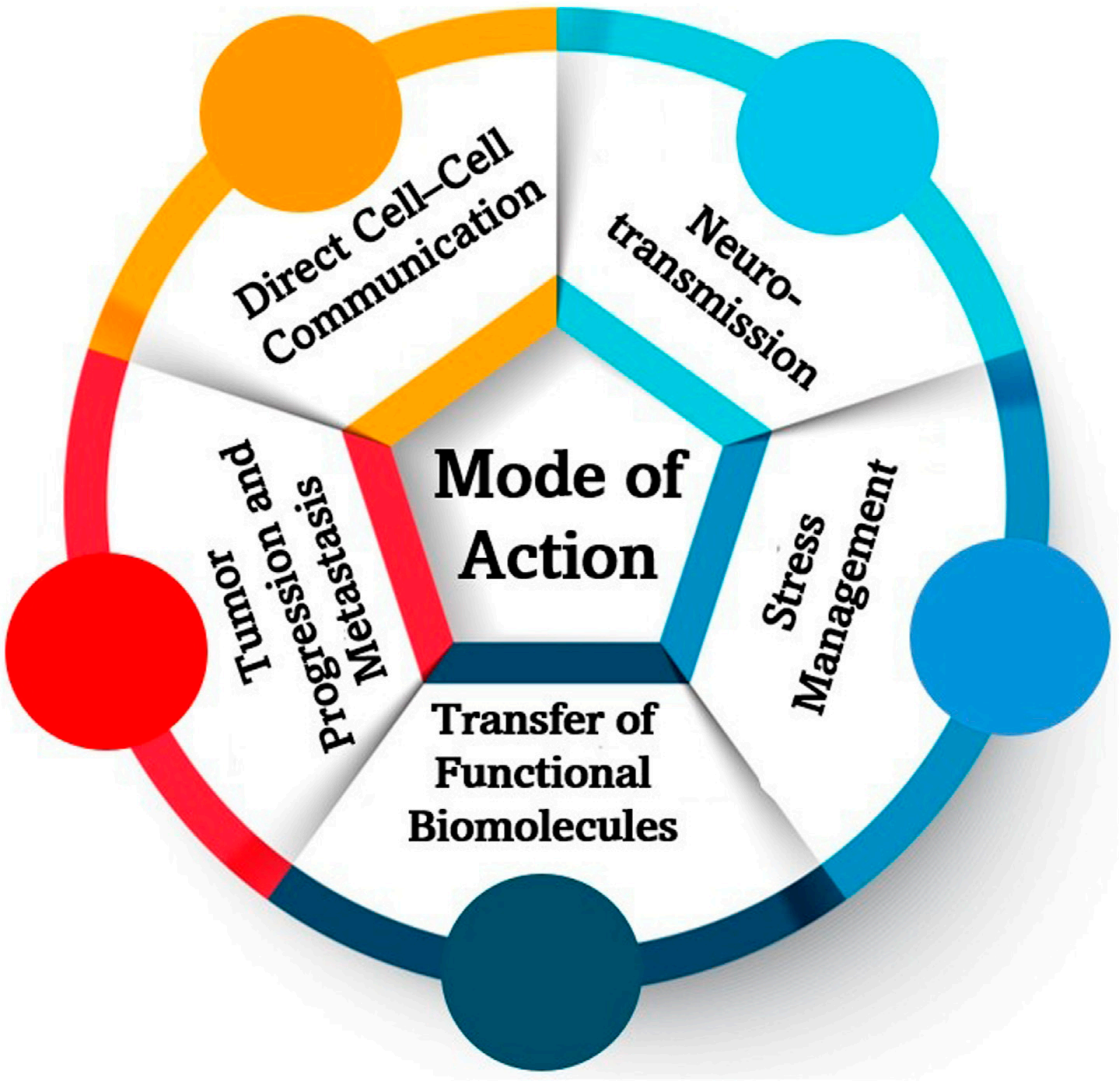
Mode of action of exosomes. Exosomes contribute to the progression or modulation of neurodegenerative diseases by influencing neuroinflammation, synaptic function, and neuronal survival by mediating intercellular communication and regulating gene expression. These actions highlight the dual role of exosomes as both biomarkers and therapeutic agents in neurodegeneration.

Exosomes are naturally occurring nanoscale vesicles derived from specific cells (Figure 6) that can facilitate drug delivery while being biocompatible and biodegradable and therefore, demonstrate a promising role in gene therapy for neurodegenerative disorders. The neuroprotective role of exosomes in CNS injuries, including traumatic brain injury (TBI), SCI, and subarachnoid hemorrhage (SAH) was studied and it was found that they help to restore cognitive abilities by facilitating neuronal repair and synaptic plasticity, thereby improving cognitive function. They are also known to regulate immune responses, minimizing neuroinflammation by suppressing the reactions. Further, they aid in neuroprotection by influencing cellular recycling mechanisms (autophagy regulation) and inhibiting cell death pathways (anti-apoptotic effect), subsequently reducing neuronal loss. They also help in maintaining BBB integrity and offering BBB protection and prevention of further damage. The key molecular mechanisms through which exosomes exert their neuroprotective effects are the microRNA (miRNA), NF-κB, PI3K/AKT, Notch1, and ERK pathways, among others (Zhang et al., 2022). Exosomes derived from glial cells can exert both neuroprotective and neurotoxic effects, depending on the cellular environment and molecular cargo they carry (Figure 7). When derived from astrocytes and oligodendrocytes, these exosomes can modulate synaptic plasticity by enhancing neuronal communication (Akbari-Gharalari et al., 2024). They accomplish this task by delivering neurotrophic factors that support synaptic signals and connections, and help in repair through macromolecules like nucleic acids and sugars. Exosomes help in the regulation of immune responses by carrying anti-inflammatory cytokines, especially by reducing neuroinflammation in conditions like AD and PD. Another mechanism of action is the transport of antioxidant enzymes that help to neutralize ROS, preventing neuronal damage. By delivering tight junction proteins, they strengthen the BBB, thereby preventing harmful substances from entering the brain (Heidarzadeh et al., 2021). Exosomes are also found to regulate autophagy by influencing cellular recycling mechanisms, and promoting the clearance of misfolded proteins associated with neurodegenerative diseases. More mechanisms of action are detailed in Figure 8. However, at times, exosomes are found to have neurotoxicity, particularly those from activated microglia that can carry pro-inflammatory cytokines, exacerbating neuroinflammation and contributing to neuronal damage. When they facilitate the intercellular transfer of misfolded proteins, such as amyloid-beta and tau, they are known to accelerate disease progression in Alzheimer’s, instead of retarding the condition. They may also contribute to further neurodegeneration as they tend to carry damaged mitochondrial components that can impair energy production. The dual role of exosome (neuroprotection and neurotoxicity) is further reinforced by the role they play in activating cell death pathways by affecting apoptotic signals, increasing neuronal apoptosis in pathological conditions (Oyarce et al., 2022).

#### 3.2.15 Nanogels

Nanogels are cross-linked polymeric networks that can swell in response to environmental stimuli, providing controlled drug release. They have high potential to serve as advanced drug delivery systems for CNS therapeutics. Due to their hydrophilic and crosslinked polymeric networks, they can offer high drug-loading capacity, controlled release, and enhanced BBB penetration (Manimaran et al., 2023). They are highly stable and compatible with biomolecules and therefore can encapsulate neuroprotective agents while maintaining their structural integrity. They enable sustained and targeted delivery, reducing side effects. They also improve drug transport across the BBB, a major challenge in CNS treatments. Nanogels are stimuli-responsive and so, they can be designed to respond to pH, temperature, or enzymatic activity, ensuring precise drug delivery. They can facilitate cellular uptake and intracellular trafficking for efficient drug absorption by neurons and glial cells. Nanogels are being explored for Alzheimer’s, Parkinson’s, and multiple sclerosis treatments as they help to reduce neuroinflammation and promote neuronal repair (Devassy et al., 2023). The neuroprotective potential of EDV can be enhanced by preparing a glutathione (GSH)-conjugated poly (methacrylic acid) nanogel, which enhances targeted brain drug delivery. This incorporation of EDV in the nanogel is supposed to ensure drug stability, controlled release, and BBB penetration, ensuring efficient neuroprotection. Studies have also demonstrated that this nanocarrier system significantly reduced oxidative stress, enhanced cognitive function, and promoted neuronal survival in ischemic conditions (Mozafari et al., 2023).

Many of the recent studies illustrate the rapid advancement in nanotechnology for neuroprotection, incorporating innovative materials and methodologies that enhance targeting, delivery, and thus offering neuroprotective functions (González et al., 2021; Waris et al., 2022; Nayab et al., 2023). Examples of some nanoparticles delivered via nanostructures to restore homeostasis in the neuronal regions in CNS, are explained in Table 2. The nanostructure type, mechanism of action, and key findings are explained. The exploration of various nanomaterials continues to hold promise for effective interventions in neurodegenerative diseases and related neurological conditions. As research progresses, the focus on safety, biocompatibility, and translation into clinical applications remains paramount.

**TABLE 2:** Types of nanoparticles that can restore homeostasis in the neuronal regions in CNS.

S. No.	Nanoparticle type	Disease model	Study type	Mechanism of action	Key findings	Citation
1	ZnO (Myco-fabricated)	Alzheimer’s (Mice)	In vivo	AChE inhibition, antioxidant	Improved memory, reduced neurotoxicity	Abd Elmonem et al. (2023)
2	Gold NPs	Parkinson’s (Rodents)	In vivo	Anti-inflammatory, BBB penetration	Reduced dopaminergic neuron loss	Biswas et al. (2025)
3	Cerium Oxide NPs	Alzheimer’s (Cell lines & Mice)	In vitro & in vivo	ROS scavenging, mitochondrial protection	Reduced amyloid burden	Biswas et al. (2025)
4	Liposomes & Exosomes	Alzheimer’s & Parkinson’s	In vivo	Targeted drug delivery	Improved bioavailability, reduced inflammation	Biswas et al. (2025)
5	Mitochondria-targeted NPs	Parkinson’s (Rodents)	In vivo	Mitochondrial biogenesis, ROS reduction	Improved motor function	Zheng et al. (2023)
6	Nanoplastics (Environmental)	Alzheimer’s & Parkinson’s	In vitro & in vivo	BBB disruption, oxidative stress	Neurotoxicity, memory impairment	Araújo et al. (2025)
7	PLGA NPs (Donepezil-loaded)	Alzheimer’s (Mice)	In vivo	Controlled release, BBB penetration	Enhanced cognition, reduced Aβ	Liu et al. (2025)
8	Silver NPs (AgNPs)	Parkinson’s (Cell lines)	In vitro	Antioxidant, anti-apoptotic	Reduced ROS, improved viability	Yadav et al. (2025)
9	Quantum Dots (CdSe/ZnS)	Alzheimer’s (Cell lines)	In vitro	Tau detection, imaging	Enabled early diagnosis	Lu et al. (2022)
10	Dendrimers (PAMAM)	Huntington’s (Rodents)	In vivo	Gene delivery, anti-inflammatory	Improved motor coordination	Krsek and Baticic (2024)
11	Iron Oxide NPs (Fe_3_O_4_)	Alzheimer’s (Mice)	In vivo	Magnetic targeting, ROS modulation	Reduced amyloid burden	Asefy, Hoseinnejhad, and Ceferov (2021)
12	Curcumin-loaded NPs	Alzheimer’s (Rodents)	In vivo	Anti-amyloidogenic, antioxidant	Reduced tau pathology	Fang et al. (2024)
13	Chitosan NPs	Parkinson’s (Cell lines)	In vitro	Dopamine delivery	Increased uptake, reduced apoptosis	Bhidayasiri (2024)
14	Graphene Oxide NPs	Alzheimer’s (Rodents)	In vivo	Anti-aggregation of Aβ	Improved spatial memory	Gao et al. (2025)
15	Exosome-mimetic NPs	ALS (Rodents)	In vivo	siRNA delivery, anti-inflammatory	Delayed motor decline	Hamdy et al. (2025)
16	Micelle-forming NPs	Alzheimer’s (Cell lines)	In vitro	Solubilization of hydrophobic drugs	Reduced tau aggregation	Kshirsagar, Patil, and Suryawanshi (2025)
17	Selenium NPs	Alzheimer’s (Rodents)	In vivo	Antioxidant, anti-apoptotic	Improved learning, reduced oxidative markers	Awad et al. (2025)
18	PEGylated NPs (BACE1 inhibitors)	Alzheimer’s (Mice)	In vivo	Enzyme inhibition, BBB targeting	Reduced Aβ production	Elawad et al. (2024)
19	TiO_2_ NPs (Green-synthesized)	Alzheimer’s (Cell lines)	In vitro	Antioxidant, anti-amyloid	Reduced Aβ aggregation	Fakhri et al. (2022)
20	ZnO NPs	skin cancer (A375 cells) and ascitic tumor cells	In vitro	Anti-amyloid activity	Induction of Aβ aggregation	Girigoswami et al. (2024)

## 4 Mechanisms of action of nanomaterials in reducing neuronal damage

**FIGURE 9:**
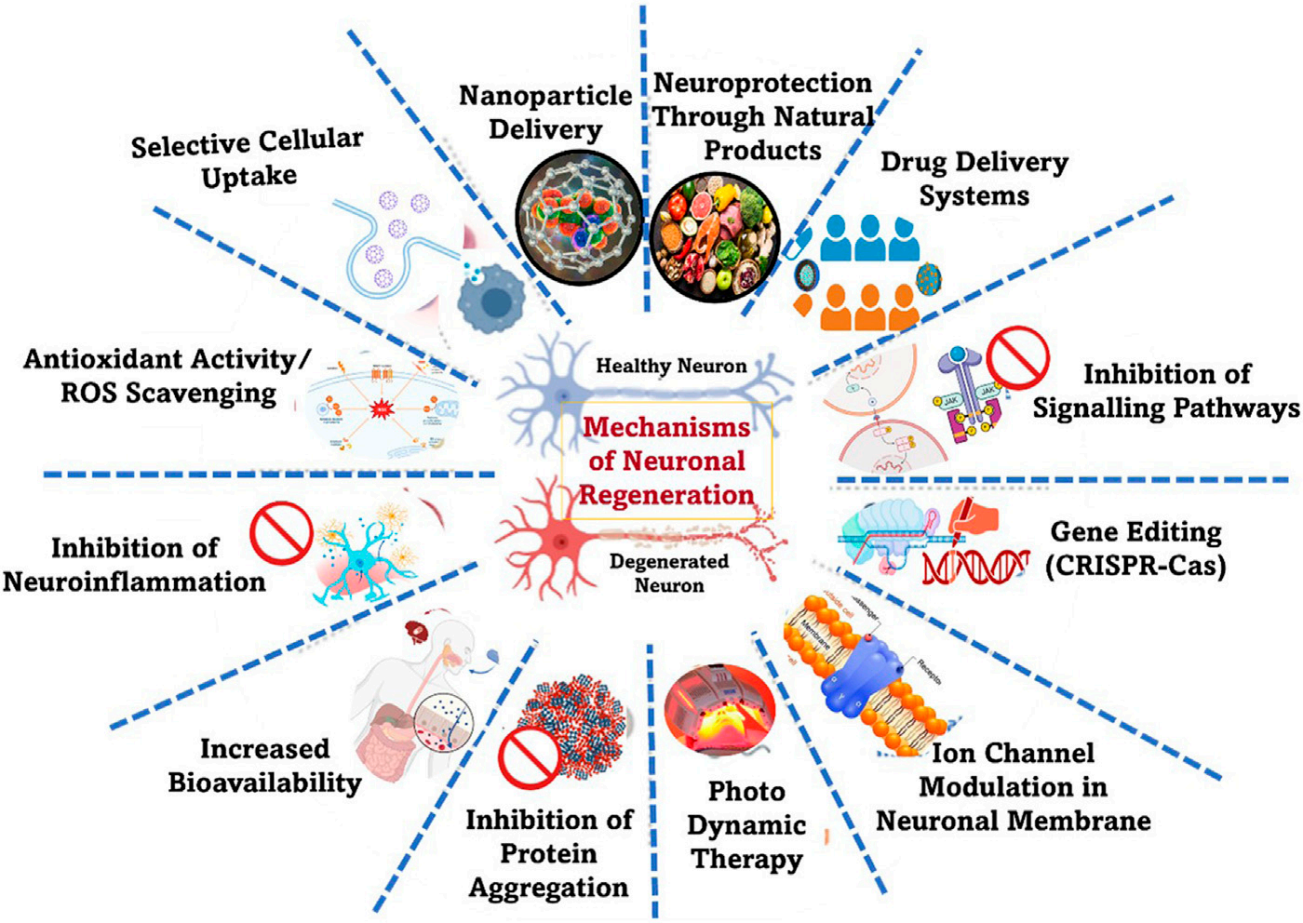
Strategies involved in neuronal damage repair. The general mode is to target key pathological processes such as oxidative stress, neuroinflammation, signalling pathways, gene editing, modulation of drug delivery system, modification of impaired cellular repair mechanisms, etc.

The mechanisms for reducing neuronal damage focus mainly on preserving neuronal function, preventing cell death, and promoting regeneration (Figure 9). Nanomaterials exhibit a variety of mechanisms of action that contribute to their potential in reducing neuronal damage and promoting neuroprotection. Their small size, large surface area, and tunable surface functionalities enable them to co-ordinate with biological systems in ways that traditional materials cannot (Bandaru et al., 2024b), and therefore these elements allow nanomaterials to cross biological barriers (even the BBB that conventional particles cannot cross). This singularity of nanomaterials allows the specific targeting of neuronal cells or pathological sites with very high precision.

Neuronal damage is caused by various mechanisms that can affect the structure and function of nerve cells in the brain and nervous system. Understanding these mechanisms that protect neurons will spark off innovative strategies for neurodegenerative diseases and brain injuries through nanotechnology, highlighting its importance in advancing neurotherapeutics. Physical injury, presence of toxic substances, lack of oxygen, etc, are known to contribute to neuronal damage (Howard et al., 2024). However, nanomaterials can exert neuroprotective and reparative effects that are central to their therapeutic potential. By scavenging ROS which are known to damage neuronal components like lipids, proteins, and DNA, they can modulate oxidative stress in neurons, resulting in reduced inflammation, and promoting neuronal regeneration, thus mitigating neuronal damage. By reducing the oxidative burden, nanoparticles such as gold and silver can prevent neuron degradation and enhance cell survival. Additionally, certain nanomaterials can activate the body’s endogenous antioxidant enzymes (e.g., superoxide dismutase (SOD) and catalase), further reinforcing cellular defense mechanisms (Jomova et al., 2024). It was observed that catalase activity is significantly lower in advanced PD cases, further exacerbating oxidative stress (Younes-Mhenni et al., 2007). Thus, nanomaterials offer a promising approach to neuroprotection by targeting these two critical defense mechanisms in neurodegenerative diseases by removing the harmful radicals that contribute to dopaminergic neuron degeneration (Nair et al., 2024). By addressing oxidative stress, nanotechnology-based interventions are positioned as highly promising therapeutic tools for managing neurodegenerative conditions, potentially slowing disease progression and improving patient outcomes.

Beyond antioxidant properties, nanomaterials also exhibit anti-inflammatory effects, playing a crucial role in preserving neuronal function (Zhu et al., 2021; Zhang et al., 2018). Inflammation in the nerve cells may affect the apoptotic mechanism leading to synaptic dysfunction. These will result in the impairment of cognitive and motor functions and, will decisively affect the quality of life of the affected person. Neuroinflammation - characterized by microglial activation and excessive cytokine release – is a major contributor to disease progression in disorders like Alzheimer’s and Parkinson’s (Lee, 2013). Nanoparticles can help inhibit microglial activation, suppress pro-inflammatory cytokines, and balance immune responses, preventing further neuronal damage. Nanomaterials can also normalize the immune response in the brain, helping to restore the balance between pro-inflammatory and anti-inflammatory signals. Further, certain nanomaterials can interfere with signaling pathways involved in the production of pro-inflammatory cytokines, decreasing their levels in the neuronal environment and reducing inflammation (Fakhri et al., 2022). Nanoparticles can also inhibit the activation of microglia, the immune cells of the CNS, reducing the release of inflammatory mediators. For example, silica nanoparticles have been shown to modulate microglial responses, thus alleviating neuroinflammation.

## 5 Targeted drug delivery using nanoparticles

Traditional drug delivery methods often result in systemic distribution, meaning that therapeutic agents spread throughout the body instead of concentrating on a specific target site. While this approach is effective for general treatments such as antibiotics or pain relief, it poses significant challenges when precision is required for conditions like neurological disorders. Further, the widespread dispersion of the drug often leads to lower therapeutic efficacy, as only a fraction of the administered dose reaches the intended site. This off-target effect could cause toxicity to the neighbouring cells in the brain that are mostly healthy and causes issues like immune suppression or cell damage (Manzari et al., 2021). Also, one major drawback of systemic drug delivery is the failure to cross BBB as the intended drug fails to reach the affected neuron, due to which the dosing requirements are also very frequent (Wu et al., 2023). Often, higher or repeated doses are needed, furthering the risk of drug resistance and cumulative toxicity. This could be problematic, particularly in case of chronic diseases like Parkinson’s, requiring long-term medication use. Targeted drug delivery (TDD), mediated through nanocarriers like liposomes, nanogels, and micelles, can address many of these limitations. Nanoparticles with their unique size, shape, surface properties, and biocompatibility, can act as nanocarriers, providing a promising platform for TDD. They can allow drugs to be encapsulated and precisely directed to diseased tissues, enhancing bioavailability. Additionally, the potential stimuli-responsive drug release of nanocarriers enables the drugs carried by the nanoparticle to be activated in response to environmental factors such as pH, temperature, or enzymatic activity, ensuring the release at the precise locality (Majumder and Minko, 2021). Other strategies of nanocarriers include ligand-based targeting, where drugs are engineered to bind specifically to receptors in diseased cells, and gene and cell-based therapies. Thus, by leveraging surface modifications, ligand-based targeting, and stimuli-responsive mechanisms, nanocarriers can improve drug circulation time, enhance cellular uptake, and facilitate controlled release, making them invaluable in treating neurodegenerative conditions like Alzheimer’s, Parkinson’s, and Huntington’s disease (Dhariwal et al., 2025; Arachchige et al., 2019). Examples of nanostructure-based therapies that are at the forefront of PD research, are explained in Table 3.

**TABLE 3:** Some nanostructure-based therapies that are at the forefront of PD research, offering innovative approaches to enhance drug delivery and target disease mechanisms.

Therapeutic agent/model	Nanostructure type	Mechanism of action	Potential benefits	References
Levodopa	Lipid nanoparticles	Enhances dopamine levels	Improved BBB penetration	Nie et al. (2021)
Dopamine Agonists	Polymeric nanoparticles	Stimulates dopamine receptors	Sustained drug release	Monge-Fuentes et al. (2021)
Curcumin	Gold nanoparticles	Anti-inflammatory & neuroprotective	Reduces oxidative stress	Palanisamy et al. (2025)
GDNF (Glial cell line-derived neurotrophic factor)	Liposomal formulations	Promotes neuronal survival	Supports dopaminergic neuron regeneration	Gash et al. (2020)
siRNA	Lipid-based nanocarriers	Gene silencing for α- synuclein aggregation	Targets PD pathology at the molecular level	Schlich et al. (2017)
Resveratrol	Polymeric micelles	Antioxidant & neuroprotective	Reduces neuroinflammation	Wang et al. (2022)
Rotigotine	Nanoemulsions	Dopamine receptor activation	Enhanced bioavailability	Arora and Gugulothu (2025)
Nilotinib	Dendrimers	Modulates autophagy	Clears toxic protein aggregates	Tocci et al. (2025)
Apomorphine	Nanocapsules	Dopaminergic stimulation	Improved drug stability	Silva et al (2021)
Graphene Oxide	Carbon-based nanocarriers	ROS scavenging & anti- inflammatory	Potential neuroprotective effects	(Kim et al. (2023)
α-Synuclein Antibodies	Lipid nanoparticles	Targets toxic protein aggregates	Reduces neurodegeneration	(Jiang et al. (2021)
Neurotrophic Factors (BDNF, NGF)	Polymeric nanoparticles	Supports neuronal survival	Enhances neuroprotection	Sandoval-Castellanos, Claeyssens, and Haycock (2021)
Omega-3 Fatty Acids	Nanoemulsions	Anti-inflammatory & neuroprotective	Improves cognitive function	Chávez-Castillo et al. (2025)
Coenzyme Q10	Liposomal formulations	Mitochondrial support	Reduces oxidative stress	Rizzardi et al. (2021)
N-Acetylcysteine (NAC)	Dendrimers	Antioxidant & glutathione booster	Protects dopaminergic neurons	Nance et al. (2017)
Peptide-based therapies	Nanocapsules	Modulates neuroinflammation	Enhances drug stability	Patel and Patel (2024)
Metal-based nanoparticles (gold, silver)	Carbon-based nanocarriers	ROS scavenging & anti- inflammatory	Potential neuroprotective effects	Verma et al. (2023), Beura et al. (2024)
Exosome-based drug delivery	Biodegradable nanoparticles	Facilitates targeted therapy	Improves drug bioavailability, neuroprotective effect	Vahab et al. (2025)
Dopaminergic cell therapy	Graphene oxide nanocarriers	Supports neuronal regeneration	Potential for disease modification	Xiong et al. (2021)
CRISPR-based gene therapy	Lipid-based nanocarriers	Genetic modulation of PD pathology	Precision-targeted treatment, neuroprotection	Akyuz et al. (2024)

### 5.1 Mechanisms of targeted drug delivery

The presence of physiological barriers, like the BBB, pose a major challenge to the delivery of traditional drugs to the brain and the delay in drug delivery will aggravate the situation due to progressive loss of neurons (Kunjiappan et al., 2021). The effective therapeutic concentrations will flounder to reach the active site in the brain, leading to poor treatment outcomes and systemic side effects. Targeted drug delivery strategies aim to enhance drug bioavailability, improve specificity, and minimize off-target reactions, making these strategies crucial for neurodegenerative disease management. Most of the recent advancements in nanotechnology and biomolecular engineering have enabled the development of nanocarriers, ligand-functionalized nanoparticles, and stimuli-responsive drug delivery systems that can efficiently cross the BBB. Further, mechanisms such as receptor-mediated transcytosis, cell-mediated transport, and intranasal drug delivery have shown promise in improving drug accumulation in affected brain regions. The mechanisms by which nanoparticles cross the BBB in the context of PD are detailed in Table 4. Such innovative strategies offer hope for more effective and personalized treatments, potentially slowing disease progression and improving patient outcomes.

**TABLE 4:** Various mechanisms by which NPs cross BBB.

Mechanism	Description	Example in PD context	References
Receptor-Mediated Transcytosis (RMT)	Ligand-functionalized NPs (e.g., transferrin, insulin) bind to endothelial receptors, triggering endocytosis and transcytosis	Transferrin-modified lipid NPs delivering siRNA targeting *SNCA* to reduce α-synuclein aggregation	Jagaran and Singh (2022)
Adsorptive-Mediated Transcytosis (AMT)	Positively charged NPs interact electrostatically with negatively charged BBB membranes, enhancing uptake especially under neuroinflammatory conditions	Cationic NPs show increased uptake in inflamed BBB regions typical of PD pathology	Hervé, Ghinea, and Scherrmann (2008)
Cell-Penetrating Peptides (CPPs)	CPPs like TAT or penetratin enable direct membrane translocation and intracellular delivery	Dual peptide-functionalized NPs targeting microglia and delivering anti-inflammatory agents in PD models	Wu and Angelova (2023)
Exploiting BBB Disruption in PD	PD-associated oxidative stress and inflammation cause localized BBB breakdown, allowing passive NP diffusion or enhanced uptake	NPs exploit compromised BBB in the substantia nigra for targeted delivery	Kim et al. (2024)
Surface Engineering & Size Optimization	NPs <100 nm with PEGylation or targeting moieties improve circulation, reduce immune clearance, and enhance BBB penetration	PEGylated PLGA NPs functionalized with angiopep-2 show enhanced BBB crossing and neuronal accumulation	Hoyos-Ceballos et al. (2020)

Nanoparticles can enhance drug delivery through several mechanisms (Zi et al., 2022; Afzal et al., 2022). Exploiting the leaky vasculature and poor lymphatic drainage in the brain, the nanoparticles could efficiently enter and accumulate more through passive targeting via EPR Effect (Subhan et al., 2021). Passive targeting of nanoparticles for drug delivery in neurodegenerative disorders relies on the natural physiological properties of the body to direct therapeutic agents to affected region of the brain. Further, nanoparticles with stealth coatings (e.g., PEGylation) can evade immune clearance, ensuring sustained drug delivery (Sheffey et al., 2022). Also, through endocytosis and cellular uptake mechanisms, neurons and glial cells can internalize nanoparticles, further facilitating the controlled release of drugs. Some studies mention that metallic magnetic nanoparticles (MMNPs) in a glioblastoma model that are ≤50 nm can reach the brain cells, while larger nanoparticles fail to cross the BBB. Even for smaller MMNPs, their accumulation is limited to areas near large brain tumor cells. However, active targeting using ligands could help in enhanced delivery (Yan et al., 2024; Rahim et al., 2021). Functionalizing the nanoparticles with antibodies, peptides, or aptamers can extend and exacerbate specific binding. Stimuli-responsive drug delivery (Dasgupta et al., 2024) enables nanocarriers designed to release drugs in response to pH, temperature, ultrasound, or enzymatic activity ensuring precise, controlled, and efficient therapeutic effects. Cell-mediated drug delivery (Santos et al., 2021; Levy et al., 2021) utilizes immune cells or exosomes to transport therapeutic agents directly to the site of action. Another mode of drug delivery depends on the transcytosis-based targeting (Yang et al., 2025),s where the nanoparticles are leveraged to transport endothelial cells (N-TECs) for active transcytosis across the vasculature in the brain and release the drug precisely at the targeted point.

### 5.2 Strategies for crossing the BBB

When it comes to the delivery of drugs to the intended site in the CNS, crossing the BBB is one of the most significant challenges. As is known, the BBB is a highly selective permeability barrier formed by endothelial cells that line the brain’s capillaries, protecting the brain from harmful substances while regulating the transport of essential nutrients (Wu et al., 2023). However, this barrier also limits the delivery of many drugs, which can be detrimental in treating neurological disorders. Consequently, various strategies have been developed to facilitate drug delivery across the BBB.

#### 5.2.1 Chemical modification and nanoparticle engineering

One of the most effective strategies for enhancing drug delivery across the BBB involves the chemical modification of therapeutic agents or the design of nanocarriers (Wu et al., 2023). This is made possible through the inclusion of lipophilic or small-molecule drugs that can readily diffuse through the BBB. Additionally, extremely small particles (like the nanoparticles) can be engineered to improve their brain-targeting capabilities. For instance, liposomes and polymeric nanoparticles when modified with specific ligands like transferrin, folate, or peptides can bind to receptors that are on the surface of endothelial cells (Mojarad-Jabali et al., 2022). These modifications will eventually facilitate receptor-mediated endocytosis, allowing nanoparticles to be internalized by the endothelial cells and subsequently releasing their cargo within the CNS. Such targeted delivery systems are known to enhance the efficacy of drugs while reducing systemic side effects.

#### 5.2.2 Disruption of the BBB

Another approach is the transitory disruption of the BBB to allow for the passage of therapeutic agents. To bring about this short and abrupt disruption, various physical and chemical methods are employed, including the use of hyperosmotic agents (e.g., mannitol) that can induce shrinkage of endothelial cells, thereby increasing permeability. Additionally, other means like focused ultrasound (FUS) combined with microbubbles are used which are known to have significant effect on opening the BBB transiently. When ultrasound is applied, the microbubbles vibrate and induce small openings in the BBB, allowing for the delivery of various therapeutic molecules, including large drugs or genetic material (Kim et al., 2023b). This is a strategy employed even for the precise targeting and controlled release of the intended drug, leading to minimal collateral damage to surrounding healthy tissue.

#### 5.2.3 Utilization of biological transport mechanisms

Leveraging the biological transport mechanisms for the delivery of drugs across the BBB is another promising strategy that involves utilizing endogenous transport systems (like the carrier-mediated transport) that naturally transport substances across the BBB. For instance, glucoproteins like glucose transporters can be exploited to deliver therapeutics that mimic glucose. Similarly, the transport of conjugated therapeutic agents can be enhanced using monoclonal antibodies or antibody fragments that target specific receptors on the BBB. The potential of exosomes and extracellular vesicles as natural vehicle systems are also explored for crossing the BBB as these explicit vesicles can be specifically engineered to carry drugs or RNA therapeutics (Ramos-Zaldívar et al., 2022). They can prove to be quite natural for transport of drugs that are generally utilised by cells to shuttle molecules across the barrier.

Even though the BBB is a highly selective membrane intended to protect the brain from toxins and invading pathogens, despite its semipermeable nature, the BBB is mostly impenetrable to most drugs, especially large or hydrophilic molecules, which restrict their entry into the brain and bloodstream. Nanoparticles have an inherent quality to overcome this challenge through several engineered mechanisms by exploiting the physiological transport pathways and disease-specific BBB alterations (Jiménez et al., 2025).

## 6 Safety assessment and risks of nanoparticles in the treatment of neurodegeneration

Targeted drug delivery, gene therapy and neuroprotection involving nanoparticles are proving to be effective in controlling PD. But the unique physicochemical properties of nanoparticles like the ultra-small size, and high surface area, along with variable surface charge is understood to pose many safety concerns (Mellor and Uchegbu, 2022). After administering the drug, NPs carrying them will easily cross the BBB and interact with the brain cells and trigger effects like oxidative stress, inflammation, or mitochondrial dysfunction. These dose-dependent reactions are also influenced by the material composition. Metal or polymers acting as nanoparticle drug can cause surface modifications, and bring about degradation kinetics (Visan et al., 2021). To avoid such reactions/effects, and to evaluate the cytotoxicity, biodistribution, and long-term accumulation of these particles in neural tissues, it is essential to conduct rigorous *in vitro* and *in vivo* testing. The lack of standardized protocols to assess aspects that are missed by the conventional toxicity assays may lead to complications as part of the interaction between the NPs and the neural cells or immune cells. To overcome this challenge, modern approaches such as adverse outcome pathways (AOPs), high-throughput screening, and organ-on-chip models are being explored to predict neurotoxicity associated with systemic routes. Additionally, advanced regulatory frameworks are implemented to address the unique performance of NPs in the biological system. This is a strategic exercise to ensure the precise characterization and reproducibility that are often overlooked in clinical translation (Tirumala et al., 2021).

## 7 Clinical trials

Clinical studies are intended to carefully assess the effectiveness of new medications. According to the U.S. Food and Drug Administration (FDA), the primary goal of Phase I trials is to determine appropriate dosage and safety, with approximately 70% of drugs progressing to Phase II where the treatment’s efficacy and its potential side effects are verified. Around 33% of these then advance to Phase III, which focuses on adverse reactions of the drug and investigate their potency (Sayyaed et al., 2023). At present, there are several clinical trials that are exploring the safety and efficacy of nanostructured therapies for neuroprotection in patients with neurodegenerative diseases. For instance, a recent Phase I trial investigated the use of polymeric nanoparticles loaded with neurotrophic factors for the treatment of ALS, targeting neuroprotection by delivering essential growth factors directly to affected motor neurons (Bondarenko and Saarma, 2021). Another promising avenue involves lipid-based nanoparticles designed to deliver siRNA targeting genes implicated in Huntington’s disease. These innovations not only enhance the specificity of treatments but also hold the potential to transform therapeutic strategies through precise modulation of gene expression. As research progresses, nanostructured technologies are set to revolutionize both the prevention and management of neurodegenerative diseases, making a significant impact in the field of neuroprotection. More details on various clinical trials in the area are represented in Table 5. Regardless of these diligent and innovative progresses, the clinical translation of nanostructured therapies face significant challenges related to toxicology, biocompatibility, regulatory hurdles, and ethical considerations (Kardani, 2024), as is seen with any other field of research. Though enhanced drug delivery and therapeutic precision is ingrained in nanomaterials, their biocompatibility is often a topic of debate among researchers that need to be thoroughly assessed to prevent cytotoxicity, immune responses, and long-term accumulation in tissues. To understand this and to ensure safe use of these therapeutic modalities, regulatory agencies impose strict guidelines. Yet the approval process is slowed down due to the lack of standardized protocols for nanoparticle characterization and toxicity testing. Additionally, questions on ethical issues arise very frequently regarding patient consent, long-term effects, and equitable access to nanomedicine. This requires transparent policies and interdisciplinary collaboration between various agencies across the globe to balance innovation with responsible implementation.

**TABLE 5:** Clinical trials exploring various strategies for neuroprotection in PD. These trials are aimed at advancing neuroprotective strategies for improving drug delivery, neuroregeneration, and disease management in PD.

National clinical trial number	Therapeutic purpose (DMT or ST)	Intervention/ therapy category	Study title	Current status (as on August 2025)
NCT06021756	ST	Ketamine/ Glutamatergic	Phase I Open-label Study of Low-dose Ketamine Infusion Treatment in Levodopa-Induced Dyskinesia in Parkinson's Disease	Active, not recruiting
NCT05979415	ST	Apomorphine/ Dopaminergic - DA agonist	Effect of Folic Acid in Levodopa Treated Parkinson's Disease Patients	Terminated
NCT05931575	DMT	Fasudil/ Kinase inhibitor	Safety, Tolerability and Symptomatic Efficacy of the ROCK-Inhibitor Fasudil in Patients with Parkinson's Disease	Active, Recruiting
NCT05931484	DMT	Gemfibrozil/ Neurotrophic factors	Study to Evaluate the Safety, Tolerability, Efficacy, and PK of FHL- 301 in Parkinson's Disease Patients	Not yet recruiting
NCT05796167	ST	Pimavanserin/ Serotonergic	Pimavanserin for Sleep in Parkinson Disease	Withdrawn
NCT05778617	DMT	Ambroxol/GBA	Ambroxol to Slow Progression in Parkinson’s Disease	Recruiting participants in the UK
NCT05709301	ST	Donepezil/ Cholinergic	Randomized Clinical Trial of Donepezil for the Treatment of Mild Cognitive Impairment in Parkinson's Disease	Not yet recruiting
NCT05677633	DMT	Sargramostim/ Anti-inflammatory	Biomarker Validation Following Sargramostim Treatment in Parkinson's Disease	Completed
NCT05635409	ST	hESC-DA progenitors/ Cell therapy	A Trial to Determine the Safety and Tolerability of Transplanted Stem Cell Derived Dopamine Neurons to the Brains of Individuals With Parkinson's Disease	Active, not recruiting
NCT05611372	ST	Rasagiline	Efficacy and Safety of Rasagiline in Prodroal Parkinson's Disease	Withdrawn (as of May 2025)
NCT05610189	ST	Tavapadon/ Dopaminergic - DA agonist	Multiple-dose Trial to Determine the Clinical Bioequivalence Between Tavapadon Tablets in Participants With Parkinson's Disease	Recruiting
NCT05589766	DMT	Nicotinamide Riboside/ Energy and mitochondria	N-DOSE: A Dose Optimization Trial of Nicotinamide Riboside in Parkinson's Disease	Completed
NCT0555118	ST	Nicergoline/ Adrenergic	Nicergoline Use in Dysphagia Patients	Not yet recruiting
NCT05830396	DMT	Small-molecule chaperone in LNPs	GREAT Trial: Ambroxol in Early PD with GBA Mutation	Ongoing
NCT05287503	DMT	Lipid nanoparticle–encapsulated Ambroxol NCT06002188	Ambroxol for Lysosomal Enhancement in PD	Completed
NCT04575259	ST	Gold nanoparticle-based imaging agent	Evaluation of AuNPs for Early PD Diagnosis via α-Synuclein Detection	Completed
NCT05681247	DMT	PLGA nanoparticles with HDAC inhibitors	SAHA-Loaded Nanoparticles for Neuroprotection in MPTP-Induced PD	Ongoing
NCT05914421	DMT	Polymeric nanoparticles for gene therapy	GBA1 Gene Therapy via Nanoparticle Delivery in PD Models	Preclinical (Translational)
NCT06002188	ST	Magnetic nanoparticles for axonal repair	Nano-Pulling for Nigrostriatal Reconnection in PD	Early Phase I
NCT05471609	ST	levodopa/carbidopa/ Dopaminergic - LD reformulation	Sustained Release Oral Formulation for Treatment of Parkinson's Disease	Suspended
NCT05266417	DMT	Insulin & Glutathione/ Energy and mitochondria	Intranasal Insulin and Glutathione as an Add-On Therapy in Parkinson's Disease	Recruiting
NCT05084365	DMT	Sulforaphane/ Antioxidant	A 6-month Study to Evaluate Sulforaphane Effects in PD Patients	Unknown Status^a^
NCT04976127	DMT	Talineuren/ Neurotrophic factors	Safety Evaluation of Intravenous Talineuren (TLN) in Parkinson's Disease-affected Patients	Active, not recruiting
NCT04932434	ST	Psilocybin / Serotonergic	Psilocybin Therapy for Depression and Anxiety in Parkinson's Disease	Completed
NCT04691661	DMT	Radotinib/ Kinase inhibitor	Safety, Tolerability, Pharmacokinetics and Efficacy Study of Radotinib in Parkinson's Disease	Recruiting
NCT03659682	DMT	Semaglutide/ GLP-1R agonist	GLP1R in Parkinson's Disease	Unknown Status^a^

ST, Symptomatic therapy - therapies focus on relieving symptoms without directly affecting the disease’s underlying mechanisms, DMT, Disease-modifying therapy - treatments designed to slow down or alter the progression of a disease, DA antagonist, dopamine agonist - a type of drug that activates dopamine receptors in the brain, mimicking the effects of dopamine, GBA, gene encoding the enzyme glucocerebrosidase (GCase) - mutations in GBA are among the most significant genetic risk factors for PD, hESC-DA, human embryonic stem cell-derived dopamine neurons - transplanting hESC-DA neurons into animal models is apparently known to restore dopamine neurotransmission and improve motor function, LD, Levodopa - a key medication used to treat Parkinson’s disease, as it helps replenish dopamine levels in the brain, GLP-1R, Glucagon-like peptide-1 receptor - drugs that mimic the natural GLP-1 hormone, which helps regulate blood sugar levels and appetite.

a Unknown Status - Study has passed its completion date and status has not been verified in more than two years.

### 7.1 Clinical relevance of nano-structured strategies in neurodegeneration

The transformative changes in interventions, leading to modifications of strategies to control diseases, have led to the integration of nano-structured blueprints into the management of neurodegenerative diseases. This shift from symptomatic relief to targeted, disease-modifying interventions has been clinically proven to offer many advantages that promptly overcome the restrictions of conventional therapies. The nanoparticles engineered to cross the BBB can precisely deliver neuroprotective agents to affected regions of the brain, thereby minimizing the widely observed side effects to enhance the therapeutic efficacy of the drug (Naqvi et al., 2020). This strategy is specifically relevant in the case of PD where the dopaminergic neuron degeneration is localized and demands site-specific intervention. On closer observation, the use of biocompatible nanocarriers like lipid-based nanoparticles or polymeric scaffolds, or even exosome-mimetic vesicles are shown to improve drug stability, cellular uptake, and sustained release profiles. Maintaining a specific therapeutic concentration in the brain over a prolonged period is crucial in chronic conditions like PD. Additionally, the integration of ligands like transferrin, lactoferrin, or peptides to these drugs is known to enhance the selectivity and stability of the drug, which further helps to reduce off-target effects and significantly improve the clinical outcomes (Padilla-Godínez et al., 2022). Documentary evidence preceding clinical studies suggests that modulation of neuroinflammation is a key contributor to disease progression (Zhang W. et al., 2023). As discussed, the long-term safety, immunogenicity, and regulatory standardization also need to be addressed before progressing to clinical translation. Even if the biocompatibility of NPs is studied extensively in cell lines (*in vitro*) and animal models (*in vivo*), human trials are essential to evaluate the biodistribution, clearance mechanisms, and potential accumulation in brain cells/tissues.

## 8 Emerging trends and challenges

A better understanding of the various aspects of neurobiology has increased the prospects of the application of nanoparticles in neurotherapeutics. Advances in targeted delivery systems and material science have paved the way for innovative trends in the field, including the development of multifunctional nanoparticles that are engineered to deliver drugs while simultaneously modulating immune responses and crossing the BBB with high precision and specificity (Emeihe et al., 2024). The incorporation of surface ligands like transferrin (Georgieva et al., 2014), lactoferrin (Singh et al., 2016), or peptides that target BBB receptors are known to enhance the uptake of the drug in the brain and minimize off-target effects. Exosome-mimicking vesicles or polymeric carriers that are derived from natural sources led to the development of biodegradable and biomimetic nanoparticles. Compared to conventional NPs, they offer improved biocompatibility and reduced immunogenicity, eliminating the safety concerns associated with synthetic nanomaterials. In addition, certain stimuli-responsive nanoparticles are found to release their payload under various pH, temperature, or enzymatic conditions. Such particles can fine-tune the drug release within the microenvironment of the disease (Parvin et al., 2025). However, translational gaps exist, especially regarding the safety of neural tissues and the long-term toxicity of nanoparticles that accumulate and potentially interfere with neuronal function. General challenges, as observed with conventional NPs, are more pronounced as recent trends are not adequately standardized to produce them in scalable amounts. The regulatory landscape is still evolving, and the provincial agreement on evaluation protocols and approval pathways is still a major challenge (Car et al., 2025). Proper execution of interdisciplinary collaboration, robust clinical trial design, and continued innovation in nanoparticle engineering will help overpower such challenges.

## 9 Conclusion

The design and application of nanostructured scaffolds support a futuristic channel/platform for neuronal growth, nurturing long-term therapeutic efficacy mediated through cell adhesion and differentiation all the while enhancing biocompatibility. Similarly, the integration of ingenious biomaterials with stem cell therapy enables a synergistic approach, improving the survival and functional recovery of dopaminergic neuron, which is crucial in neurodegeneration management. Furthermore, the recent research on the integration of electrical stimulation via nanoscale devices is paving the way for precision-targeted neuromodulation (Alipour et al., 2025), enhancing synaptic activity and restoring motor function in PD patients. These innovations collectively contribute to a paradigm shift in neurodegenerative disease treatment, ensuring effective drug delivery, improved cellular responses, and optimized neuroprotective outcomes. By leveraging these nanostructured technologies, researchers are moving closer to clinically viable solutions, offering hope for enhanced patient outcomes and long-term disease management. It is evident from preclinical studies that nanoparticles have promising outcomes in targeted drug delivery, leading to neuroprotection, and the modulation of neuroinflammation is effective under the application of nanostructures. Clinical translation of many of these research remains limited by challenges including biocompatibility, long-term safety, and regulatory standardization with regard to nanoparticles. By including clinical trial data in translational therapeutics along with strategies for patient stratification, a real-world therapeutic outcome can be achieved. This will enhance the scientific depth of such a research and positions nano-therapeutics as a most viable alternative to the ever-evolving landscape of neurodegenerative disease management.
